# Regulation of angiotensin II actions by enhancers and super-enhancers in vascular smooth muscle cells

**DOI:** 10.1038/s41467-017-01629-7

**Published:** 2017-11-13

**Authors:** Sadhan Das, Parijat Senapati, Zhuo Chen, Marpadga A. Reddy, Rituparna Ganguly, Linda Lanting, Varun Mandi, Anita Bansal, Amy Leung, Selena Zhang, Ye Jia, Xiwei Wu, Dustin E. Schones, Rama Natarajan

**Affiliations:** 1 Department of Diabetes Complications and Metabolism, Diabetes Metabolism Research Institute, Beckman Research Institute of City of Hope, Duarte, CA 91010 USA; 2 Department of Molecular and Cellular Biology, Beckman Research Institute of City of Hope, Duarte, CA 91010 USA

## Abstract

Angiotensin II (AngII) promotes hypertension and atherosclerosis by activating growth-promoting and pro-inflammatory gene expression in vascular smooth muscle cells (VSMCs). Enhancers and super-enhancers (SEs) play critical roles in driving disease-associated gene expression. However, enhancers/SEs mediating VSMC dysfunction remain uncharacterized. Here, we show that AngII alters vascular enhancer and SE repertoires in cultured VSMCs in vitro, ex vivo, and in AngII-infused mice aortas in vivo. AngII-induced enhancers/SEs are enriched in binding sites for signal-dependent transcription factors and dependent on key signaling kinases. Moreover, CRISPR-Cas9-mediated deletion of candidate enhancers/SEs, targeting SEs with the bromodomain and extra-terminal domain inhibitor JQ1, or knockdown of overlapping long noncoding RNAs (lncRNAs) blocks AngII-induced genes associated with growth-factor signaling and atherosclerosis. Furthermore, JQ1 ameliorates AngII-induced hypertension, medial hypertrophy and inflammation in vivo in mice. These results demonstrate AngII-induced signals integrate enhancers/SEs and lncRNAs to increase expression of genes involved in VSMC dysfunction, and could uncover novel therapies.

## Abstract (web summary)

The repertoire of tissue-specific distal regulators of gene transcription enhancers defines homeostasis or disease. Here, the authors reveal the enhancer and super-enhancer signature of vascular smooth muscle cells under normal and angiotensin II stimuli, providing new insight into the transcriptional regulation of vascular pathologies.

## Introduction

Activation of aortic vascular smooth muscle cells (VSMCs) by the pro-inflammatory and pro-atherogenic growth factor (GF) angiotensin II (AngII) is a key event in the development of cardiovascular diseases (CVDs), such as hypertension and atherosclerosis^1,2^. AngII induces VSMC contraction, proliferation, and hypertrophy in the blood vessel wall. AngII actions are mediated through its type-I (AT_1_R) and type-II receptors (AT_2_R)^3^. Most of the adverse effects of AngII occur through the activation of AT_1_R and downstream signal-transduction pathways. Thus, angiotensin-converting enzyme (ACE) inhibitors (which block the conversion of AngI to AngII) and AT_1_R blockers are widely used for the treatment of hypertension and atherosclerotic CVD^4^. AngII induces the expression of pro-fibrotic and pro-inflammatory genes in VSMCs, including plasminogen activator inhibitor-1 (*Serpine1*), Interleukin-6 and Interleukin-18 (*Il6* and *Il18*), Chemokine (c-c) motif ligand 2 (*Ccl2*), collagen and fibronectin^1,5–9^. Our recent studies show that AngII induces expression of not only microRNAs^10^, but also long noncoding RNAs (lncRNAs) in VSMCs, some of which modulate VSMC proliferation^11^. AngII regulates gene expression via activation of transcription factors (TFs) such as AP1, NF-κB, and ETS1, and co-operating epigenetic mechanisms that include increased histone H3 lysine 9/14 acetylation (H3K9/14ac) as well as other histone modifications at target gene promoters^8,11^. However, the genome-wide changes in distal regulatory regions (enhancers) triggered by AngII and their epigenetic functions in AngII-mediated gene regulation and vascular actions remain unclear.

Cellular gene expression is a complex process involving regulation by TFs acting at *cis*-regulatory elements in proximal gene promoters and distal regulatory elements situated far away from promoters, i.e., enhancers^12^. Enhancers are more cell/tissue-specific than promoters and can therefore provide critical regulatory information^13,14^. Mammalian cells harbor thousands of transcriptional enhancers that control specific gene expression patterns^13,15^. Enhancer activity is determined by multiple TF binding and enhancer-specific histone modification marks^16^. As per current consensus, monomethylated histone H3 at lysine4 (H3K4me1) marks inactive, active and poised enhancers, whereas acetylated histone H3 at lysine27 (H3K27ac) distinguishes active enhancers^14,17–19^. Moreover, large clusters of enhancers, known as super-enhancers (SEs), characterized by high enrichment of transcriptional coactivators, TFs and chromatin regulators, determine cell fate by controlling cell type-specific gene expression patterns and drive expression of genes implicated in disease^20–22^. The transcriptional coactivator bromodomain protein 4 (BRD4) belonging to the family of bromodomain and extra-terminal domain (BET) proteins can function as a scaffold for TFs at promoters and SEs. It is a positive regulator of SE-mediated transcription in cancer, autoimmune diseases, as well as atherosclerosis, acting at many canonical pro-inflammatory endothelial genes^22,23^. Recent studies also show that co-operation between enhancer-associated lncRNAs and TFs is a key mechanism driving cell type-specific gene expression^24^. In this study, we examine for the first time the repertoire of VSMC-specific enhancers/SEs and their functional roles in AngII-induced gene expression.

Here, we profile the AngII-regulated enhancers/SE repertoires in rat VSMCs (RVSMCs) and show that enhancers/SEs drive AngII-regulated gene expression in RVSMCs, rat aortas, and aortas of AngII-infused mice. We demonstrate the functionality of these enhancers/SEs in AngII-induced gene expression using approaches such as genome editing, treatments with the BET/bromodomain inhibitor JQ1, or RNAi-mediated knockdown of lncRNAs overlapping enhancers. Moreover, we show that blocking SE formation using JQ1 ameliorates AngII-related CVD phenotypes such as hypertension, medial hypertrophy, and inflammation in vivo in mice. Together, the results reveal novel functional roles of enhancers/SEs in epigenetic mechanisms of AngII actions, which in turn could yield valuable new information for therapeutic intervention in CVDs.

## Results

### AngII alters the enhancer repertoire of VSMCs

**Fig. 1:**
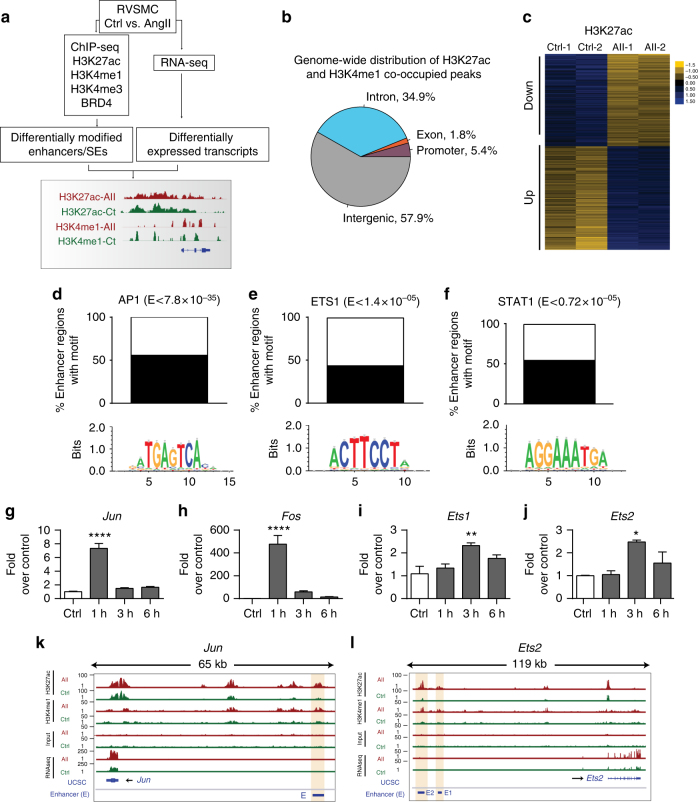
AngII-regulated TFs involved in VSMC enhancer repertoire. **a** Schematic depicts strategy employed to identify AngII-induced enhancers and SEs in RVSMCs using high-throughput ChIP-seq and RNA-seq data. **b** Genomic distribution of co-occupied H3K27ac and H3K4me1-enriched regions. **c** Heatmaps showing the differential enrichment of H3K27ac at enhancers (1541 upregulated and 1328 downregulated) in Control (Ctrl) and AngII (AII)-treated RVSMCs compared to 5201 unaffected enhancers (fold change ≤ 1.05). H3K27ac enrichment in form of log2 ChIP vs. input across four samples (as indicated on top of the column) were mean centered at each enhancer and visualized by heatmap with yellow indicating below average and blue above average. **d**–**f** Bar graphs showing percentage of upregulated enhancers enriched with indicated signal-dependent TF binding motifs after AngII treatment (H3K27ac increased by ≥2 fold, *q* < 0.05). E is Bonferroni-adjusted *P-*value. **g**–**j** Bar graphs represent changes in mRNA levels of indicated TFs upon AngII treatment (1–6 h). Gene expression normalized to *Ppia* is expressed as Fold over control. Mean + SEM, *P < 0.05; **P < 0.01; ****P < 0.0001, *n = *3. Significance was calculated using one-way ANOVA, Dunnett’s multiple comparisons test comparing the means of each time point to the mean of Control (Ctrl). **k**, **l** ChIP-seq and RNA-seq tracks showing enhancers for indicated TFs. Blue horizontal bars-enhancers (E), Orange vertical bar-H3K27ac signal

In order to characterize the AngII-regulated enhancers that might be involved in AngII-mediated gene expression in RVSMCs, we performed genome-wide ChIP-seq analysis using antibodies recognizing H3K4me1 for candidate enhancers and H3K27ac for active enhancers^18^ (Fig. 1a). ChIP-seq was performed in two biological replicates from Control (untreated) and AngII (100 nM)-treated RVSMCs. All samples yielded >25 million reads that passed ChIP-seq quality control measures by FastQC^25^. Peaks were called for each histone modification data set using MACS2 (see Methods). Cluster analysis and scatter plots of read counts showed that two biological replicates of ChIP-seq data for control and AngII-treated samples were consistent with each other and well correlated (Supplementary Fig. 1a–i). H3K27ac and H3K4me1 peaks were mostly present in intronic and intergenic regions, with only a small proportion in the promoter regions (Supplementary Fig. 2a, b), in accordance with the reported distribution of enhancer marks in other cell types^18,26^. Next, we identified regions co-occupied by H3K27ac and H3K4me1 as they would represent *bona fide* functional enhancers^18^. As expected, the genomic distribution of the H3K27ac and H3K4me1 co-occupied regions was mainly in intergenic regions, as with the individual enhancer marks (Fig. 1b).

Since H3K27ac and H3K4me1 marks are also enriched at other regulatory elements, such as promoters, we filtered out H3K27ac/H3K4me1 co-occupied regions overlapping promoters of RefSeq genes (±1 kb from transcription start sites/TSS), exons of known genes, and H3K4me3-enriched peaks (potential promoters) (Supplementary Fig. 3 and Methods). Differentially enriched enhancers (Control vs. AngII) were then identified for H3K27ac (Fig. 1c, see also Methods), which showed that AngII increased H3K27ac levels at 1541 and decreased at 1328 enhancers in RVSMCs (Fig. 1c), henceforth referred to as upregulated and downregulated enhancers, respectively, compared to 5201 unaffected enhancers. These results show that AngII leads to extensive genome-wide alterations in the enhancer landscape in VSMC that might have significant ramifications in AngII-mediated gene regulation.

### TF binding sites enriched at AngII-regulated enhancers

To identify candidate TFs regulating the distinct AngII-induced enhancer landscape in RVSMCs, we performed de novo motif discovery analysis (see Methods). H3K27ac enhancers upregulated by AngII were enriched for the AP1, ETS, and STAT TF binding sites (Fig. 1d–f). Notably, several of these TFs have been previously implicated in AngII-mediated gene transcription at promoters^27–30^, but their enhancer roles are unknown. Strikingly, more than 60% of the H3K27ac AngII-upregulated enhancers contained AP1 binding sites, underscoring the importance of enhancer AP1 in AngII-induced gene regulation. We next investigated whether these TFs are regulated by AngII at the mRNA level. RT-qPCR analysis showed significant upregulation of *c-jun* and *c-fos* (AP1) within 1 h of AngII treatment, whereas *Ets1* and *Ets2* were upregulated after 3 h (Fig. 1g–j). Interestingly, our ChIP-seq data show that several enhancers flanking *Jun* and *Ets2* were enriched with H3K27ac after AngII stimulation (3 h), indicating potential regulation of these TFs by AngII-upregulated enhancers (Fig. 1k, l). CDX2 and FOXL1 motifs were enriched in H3K27ac-downregulated enhancers (Supplementary Fig. 4).

In-depth data analysis revealed that several H3K27ac occupied enhancers were absent in control data sets and gained only upon AngII treatment (de novo gained enhancers, Supplementary Fig. 5a). Conversely, some enhancers exhibited high H3K27ac enrichment even before stimulation, and reduced significantly upon AngII treatment indicating loss of enhancer activity (Supplementary Fig. 5b). In total, we found 265 de novo gained enhancers and 201 lost enhancers (fc ≥ 2 and *q* < 0.05; Benjamini–Hochberg (BH) adjusted). Interestingly, we again observed enrichment of AP1 motifs in addition to FOXC1 in gained (de novo) enhancers (Supplementary Fig. 5c, d). For lost enhancers, we found enrichment of binding sites for TFs CDX2, FOXL1, and LIN54 (Supplementary Fig. 5e–g). Taken together, these results indicate that specific TFs are involved in the formation of distinct AngII-regulated enhancer landscapes in RVSMCs, entailing significant contribution of the epigenome and these TFs in shaping the AngII-specific response in VSMCs.

### AngII-induced enhancers regulate expression of nearby genes

**Fig. 2:**
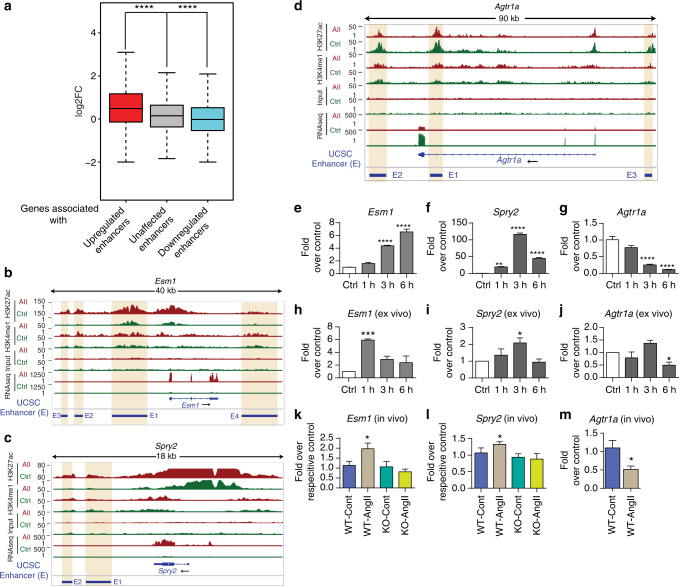
AngII-induced enhancers regulate expression of nearby genes. **a** Box plot showing genes associated with upregulated (red), unaffected (gray), and downregulated (blue) enhancers. ****P < 0.0001, using unpaired two-tailed *t*-test. **b**–**d** Profiles of H3K27ac and H3K4me1 signals for VSMC-specific enhancers around nearby differentially expressed genes *Esm1* (**b**), *Spry2* (**c**), and *Agtr1a* (**d**). Each data track shown is on the same scale for both Control and AngII samples. Differentially regulated enhancers have been highlighted with vertical orange bars. Blue horizontal bars indicate enhancers (E). **e**–**g** Bar graphs showing validation of AngII-induced expression of *Esm1* (**e**), *Spry2* (**f**), and *Agtr1a* (**g**) in RVSMCs (in vitro). **h**–**j** Bar graphs showing validation of AngII-induced expression of indicated genes in ex vivo treated rat aortas and **k**–**m** bar graphs showing validation of AngII-induced expression of indicated genes in vivo in aortas from WT or AT_1_RKO (KO) mice infused with PBS (Cont) or AngII. Gene expression was normalized to *Ppia* and expressed as Fold over control. Mean + SEM; *n* = 3 independent experiments (for **e**–**g**); *n* = 3 rat aortas (for **h**–**j**). *P < 0.05; **P < 0.01; ***P < 0.001; ****P < 0.0001, using one-way ANOVA, Dunnett’s multiple comparison. For **k**–**m**, *n = *6 mouse aortas, *P < 0.05, using unpaired two-tailed *t*-test

Expression of proximal or nearby genes is affected by the presence of enhancer elements^31^ that tend to occur as clusters proximal to the neighboring genes they regulate^26^. To further elucidate the relationships between AngII-mediated transcriptional, epigenomic, and enhancer networks in RVSMCs, we combined our current RVSMCs enhancer catalog (H3K27ac ChIP-seq data) with our published RNA-seq data (GEO #GSE38056)^11^ (Fig. 1a). Integration of these two data sets showed that putative upregulated enhancers were associated with 699 differentially expressed “nearby genes” (~500 kb proximal) in AngII-treated vs. untreated VSMCs (see Methods). Differentially enriched enhancers were assigned to their nearby RefSeq genes that showed differential expression in response to AngII, based on the criteria that their TSS were within its ±250 kb flanking regions. Overall, 35.7% of AngII-regulated genes were enhancer-associated. Genes associated with upregulated enhancers showed significantly increased expression, and downregulated enhancers showed statistically significant downregulation, compared to genes associated with unaffected enhancers (Fig. 2a). Moreover, the change in expression of enhancer-associated AngII-regulated genes positively correlates with the change in H3K27ac enrichment at corresponding enhancers (Supplementary Fig. 6).

To further characterize changes occurring at AngII-induced enhancers, we analyzed the putative enhancer regions for three candidate AngII-induced genes in VSMCs identified by RNA-seq (Fig. 2b–d). We examined two representative AngII-upregulated genes, namely, endothelial cell-specific molecule1 (*Esm1*) and Sprouty Homolog2 (*Spry2*), that are involved in VSMC growth, proliferation and inflammation^32,33^, and one AngII-downregulated gene, AngII Receptor Type1a (*Agtr1a*), which mediates AngII signaling involved in hypertension and CVD^34^. We found AngII-induced enrichment of H3K27ac at several enhancers near upregulated *Esm1* (Fig. 2b) and *Spry2* (Fig. 2c) genes. In contrast, H3K27ac signals at enhancers near downregulated *Agtr1a* decreased after AngII treatment (Fig. 2d). These putative enhancers are denoted as blue blocks and H3K27ac peaks are highlighted as vertical orange shaded boxes. These representative data suggest correlations between the H3K27ac enrichment at enhancers and the expression levels of nearby genes indicated in Fig. 2b–d.

To further investigate the relevance of these observed AngII-induced alterations in enhancer levels to the expression of the corresponding genes, we first validated the expression of these genes in RVSMCs treated in vitro with AngII. Both *Esm1* and *Spry2* levels were significantly upregulated (Fig. 2e, f), whereas *Agtr1a* levels were significantly reduced within 3 h of AngII treatment (Fig. 2g). Moreover, treatment of rat aortas ex vivo with AngII also increased *Esm1* and *Spry2* expression within 1 and 3 h, respectively (Fig. 2h, i), whereas *Agtr1a* expression decreased within 6 h (Fig. 2j). Further, we explored the in vivo relevance using aortas from wild-type (WT) and AT_1_R knockout mice (AT_1_RKO) infused with PBS (Cont) or AngII. Expression for *Esm1* and *Spry2* significantly increased (Fig. 2k, l), whereas *Agtr1a* expression decreased (Fig. 2m) in aortas from AngII-infused WT mice compared with PBS-infused control WT mice. Furthermore, *Esm1* and *Spry2* levels were not altered in aortas from AT_1_RKO-control and AT_1_RKO-AngII-infused mice, supporting the importance of AT_1_R in AngII-mediated gene expression (Fig. 2k, l). Altogether, these data clearly indicate that AngII regulates key genes in VSMCs through possible modulation of their associated enhancers.

### Validation of AngII-regulated enhancer regions in RVSMC

**Fig. 3:**
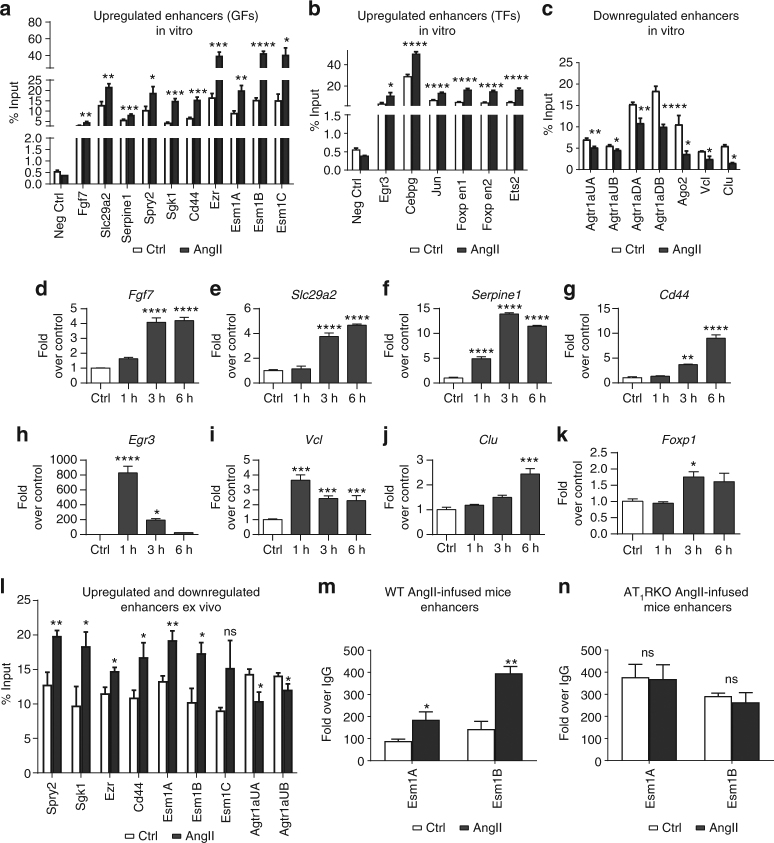
Validation of enhancer regions and expression of AngII-induced nearby genes. **a**, **b** Bar graphs represent H3K27ac enrichment at upregulated enhancers near indicated GF signaling (**a**) and TF (**b**) genes in Control (Ctrl) or AngII-treated RVSMCs. **c** H3K27ac enrichment at downregulated enhancers near indicated genes in RVSMCs untreated or treated in vitro with AngII. **d**–**k** Enhancer-associated nearby gene expression was validated in RVSMCs treated with AngII for 1–6 h in vitro. Gene expression was normalized to *Ppia* and expressed as Fold over control. Mean + SEM; *P < 0.05; **P < 0.01; ***P < 0.001; ****P < 0.0001 vs. Ctrl; *n* = 3, using one-way ANOVA, Dunnett’s multiple comparison. **l** H3K27ac enrichment at upregulated and downregulated enhancers near indicated genes in rat aortas untreated (Ctrl) or treated ex vivo with AngII. **m**, **n** H3K27ac enrichment at upregulated enhancers near indicated genes in aortas from vehicle (Ctrl) or AngII-infused WT and AT_1_RKO mice. H3K27ac enrichment is represented as Percent Input (**a**–**c**, **l**) or fold enrichment over IgG (Fold over IgG) pulldown (**m**, **n**). For **l** three rat aortas, and for **m**, **n** four mouse aortas were pooled to perform the ChIP assays. **a**–**c** and **l**–**n** Mean + SEM; *P < 0.05; **P < 0.01; ***P < 0.001; ****P < 0.0001 vs. Ctrl; *n* = 3, using unpaired two-tailed *t*-test

Next, to validate the potential enhancer regions identified from the ChIP-seq data sets, we performed follow-up chromatin immunoprecipitation (ChIP) assays with H3K27ac antibodies. ChIP-qPCRs were performed on several randomly selected AngII-regulated enhancers associated with differentially expressed genes (designated by the name of their nearby differentially expressed genes). Results confirmed upregulation of H3K27ac signals at enhancers associated with several GF signaling genes (Fig. 3a) and TFs (Fig. 3b) induced by AngII in RVSMCs. Similarly, we confirmed the inhibition of H3K27ac signals at seven potential enhancers associated with genes downregulated in AngII-treated RVSMCs (Fig. 3c). Using RT-qPCR, we also validated AngII-induced expression of 14 of these enhancer-associated GF-signaling genes in in vitro treated RVSMCs (Fig. 3d–k and Supplementary Fig. 7a–f). Note that genes near downregulated enhancers do not always show downregulated expression, e.g., *Vcl*, *Clu* (Fig. 3c vs. Fig. 3i, j). We next validated (by ChIP-qPCRs) the upregulated and downregulated enhancers in rat aortas treated ex vivo with AngII (Fig. 3l). We also validated the differential regulation of two of these enhancer regions for the *Esm1* gene in vivo using aortas from Control (PBS) and AngII-infused WT and AT_1_RKO mice. Both enhancers for *Esm1* (Esm1A and Esm1B) were upregulated in AngII-infused WT mice compared to Control (Fig. 3m) and, importantly, neither were upregulated in AngII-infused AT_1_RKO mice (Fig. 3n). Together, these results validate the ChIP-seq data for AngII-induced formation of specific enhancers in in vitro, ex vivo and in vivo model systems, and expression of nearby genes in VSMCs.

**Fig. 4:**
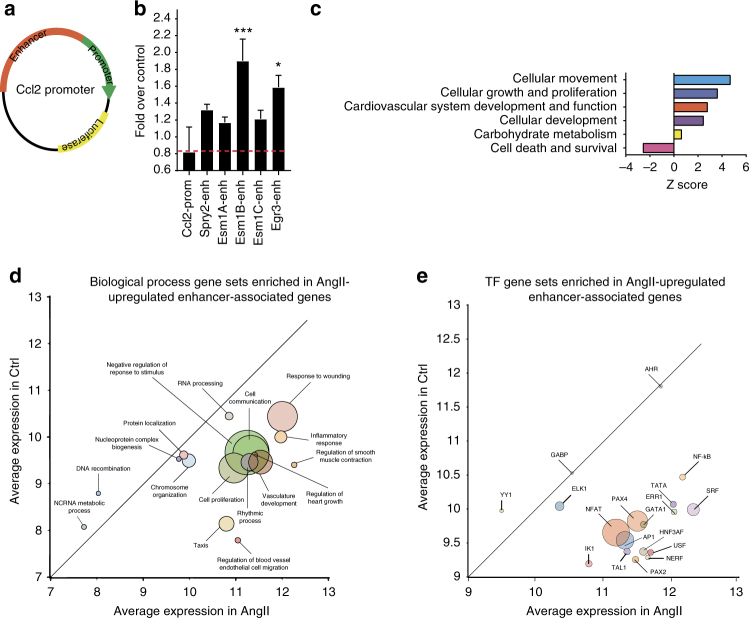
AngII-regulated enhancers are associated with VSMC phenotypes. **a** Schematic for the enhancer reporter construct with firefly luciferase gene under the control of *Ccl2* promoter. Potential enhancers (Enhancer) of indicated nearby genes were cloned upstream of the *Ccl2* promoter. **b** RVSMCs were co-transfected with *Ccl2* promoter (Ccl2-Prom) or indicated enhancer (enh) reporters and control Renilla luciferase plasmid. RVSMCs were treated ± AngII and firefly luciferase activity normalized with Renilla luciferase was expressed as Fold over control. Mean + SEM; *P < 0.05; ***P < 0.001 vs. control Ccl2-Prom; *n* = 4, using one-way ANOVA, Dunnett’s multiple comparison. **c** IPA of nearby genes associated with upregulated enhancers. **d** GSEA analysis of genes associated with upregulated enhancers. Significant biological process gene sets (empirical *P* < 0.05) below the diagonal line are upregulated, while those above or on the diagonal line are downregulated. The size of the circle is proportional to the number of significantly altered genes (0.5 < log2FC < −0.5) within that pathway. **e** Significant TF gene sets (empirical *P* < 0.05) below the diagonal line are upregulated, while those above or on the diagonal line are downregulated. The size of the circle is proportional to the number of significantly altered genes (0.5 < log2FC < −0.5) within that pathway

We next assessed the functional significance of H3K27ac-upregulated enhancers by examining whether they were capable of stimulating AngII-dependent transcription. For this, we cloned putative *Spry2*, *Esm1*, and *Egr3* enhancers upstream of the *Ccl2* promoter expressing a luciferase reporter gene (Fig. 4a). These enhancer reporter plasmids were transiently transfected into RVSMCs and luciferase activity in cell lysates determined 48 h post transfection ± AngII treatment. AngII significantly increased activity of Esm1B and Egr3 enhancers compared to the *Ccl2*-promoter alone without enhancer (Fig. 4b). These results reinforce the correlation between H3K27ac enrichment and enhancer activity in response to AngII.

### AngII-induced enhancers regulate VSMC phenotype

We next sought to uncover the putative functions of AngII-regulated enhancers in key AngII-regulated VSMC phenotypes. First, we performed in silico network analysis with Ingenuity Pathway Analysis (IPA) to determine the biological functions associated with differentially expressed genes nearby upregulated enhancers. The results revealed enrichment of diverse cellular functions, including cell death and survival, cellular development, carbohydrate metabolism, cardiovascular development and functions, cellular movement, growth and proliferation (Fig. 4c). In addition, Gene Set Enrichment Analysis (GSEA)^35^ showed upregulation of related biological processes such as regulation of smooth muscle contraction, rhythmic processes, cell proliferation, vasculature development, heart growth and inflammatory response in the AngII-treated condition (Fig. 4d). Furthermore, promoters of genes associated with upregulated enhancers contained motifs for SRF, NF-κB, AP1, NFAT, among others (Fig. 4e).

Additionally, we used the Genomic Regions Enrichment of Annotations Tool (GREAT) tool^36^ to reaffirm these phenotypes. AngII-upregulated enhancers showed association with biological processes related to GF-signaling (protein phosphorylation), metabolism, cell growth and disease phenotypes such as heart disease and inflammation (Supplementary Fig. 8a, b). In contrast, downregulated enhancers showed association with several biological processes related to cardiovascular processes, and phenotypes related to type-2 diabetes and its vascular complications (Supplementary Fig. 8c, d). Together, these in silico data indicate that AngII-regulated enhancers in VSMCs are associated with vascular disease-related phenotypes.

### Enhancer-associated lncRNAs regulate AngII-induced genes

**Fig. 5:**
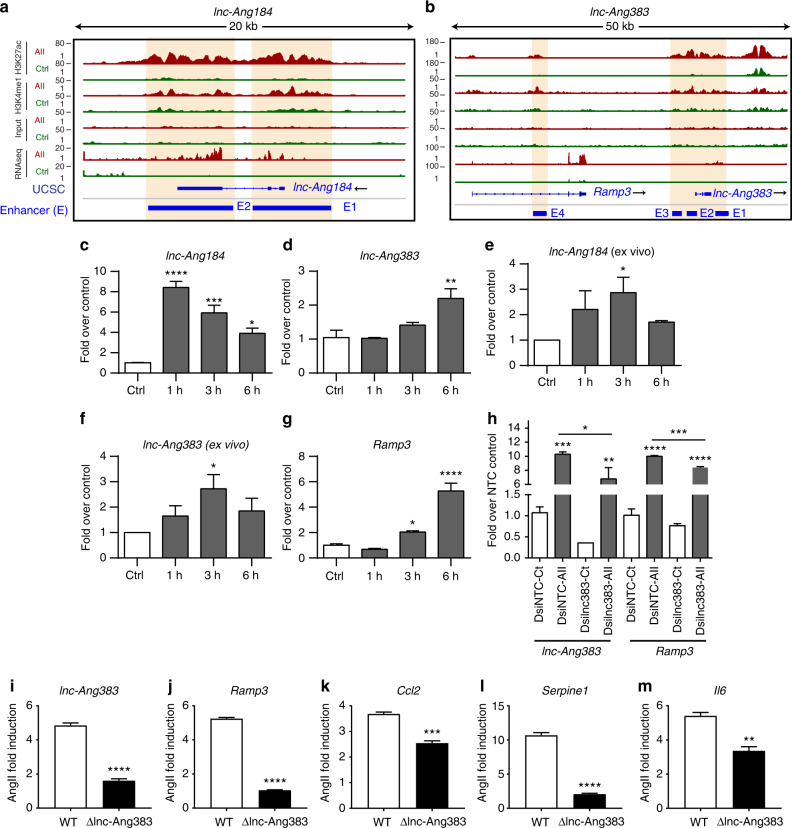
lncRNAs overlapping enhancers regulate VSMC gene expression. **a**, **b** Profiles of H3K27ac and H3K4me1 signals for VSMC-specific enhancers overlapping lncRNAs; *lnc-Ang184* and *lnc-Ang383*. Each data track for control and AngII is shown on the same scale for both Control and AngII. Orange vertical boxes indicate H3K27ac signals at differentially upregulated enhancers. Blue bars represent enhancers (E). **c**–**f** Bar graphs represent changes in the expression of *lnc-Ang184* and *lnc-Ang383* in RVSMCs treated in vitro (**c**, **d**) and rat aortas treated ex vivo (**e**, **f**) with AngII for indicated times. Gene expression was normalized to *Ppia*. Mean + SEM; *n* = 3 independent experiments for **c**, **d**, and *n* = 4 rat aortas for **e**, **f**; *P < 0.05; **P < 0.01; ***P < 0.001; ****P < 0.0001, using one-way ANOVA, Dunnett’s multiple comparisons test. **g** RT-qPCR analysis of AngII-induced *Ramp3* expression in RVSMCs at indicated times. Gene expression was normalized to *Ppia*. Mean + SEM; *n* = 3 independent experiments, *P < 0.05; ****P < 0.0001, using one-way ANOVA, Dunnett’s multiple comparisons test. **h** Expression of AngII-induced (3 h) *lnc-Ang383* and *Ramp3* in RVSMCs transfected with DsiRNAs targeting *lnc-Ang383* (Dsilnc383) or control DsiNTC. Gene expression was normalized to *Ppia* and results expressed as fold over siNTC control. Mean + SEM; *n* = 3 independent experiments, *P < 0.05; **P < 0.01; ***P < 0.001; ****P < 0.0001, using one-way ANOVA, Tukey’s multiple comparisons test. **i**, **j** Bar graphs showing AngII-induced expression of *lnc-Ang383* and *Ramp3* in WT and *lnc-Ang383* deleted cells (generated by CRISPR-Cas9 editing). **k**–**m** AngII-induced expression of pro-inflammatory genes *Ccl2*, *Serpine1*, and *Il6* in RVSMCs with *lnc-Ang383* deletion (ΔlncAng-383) and WT cells. Gene expression was normalized to *Ppia*. Mean + SEM; *n* = 4 independent experiments, **P < 0.01; ***P < 0.001; ****P < 0.0001, using unpaired two-tailed *t*-test (**i**–**m**)

Recent studies suggest a strong correlation between lncRNAs overlapping enhancers and expression of the associated protein coding gene^24^. The expression of lncRNAs overlapping regulatory elements is also a mark of active enhancers^37–39^. We therefore examined the potential association of our VSMC lncRNAs^11^ with enhancers identified in our current analysis. We found that 211 enhancers overlapped with 170 lncRNAs expressing genomic regions (empirical *P-*value *P* < 0.05–*P* < 0.0001). We then evaluated whether enhancer-associated lncRNAs have any role in regulating the expression of AngII-induced nearby genes. As representative examples, we chose two AngII-induced lncRNAs *lnc-Ang184* and *lnc-Ang383* that overlapped enhancers (Fig. 5a, b). We first validated the AngII-induced upregulation of these lncRNAs by RT-qPCR in RVSMCs (Fig. 5c, d) and in rat aortas treated ex vivo (Fig. 5e, f). Interestingly, *Ramp3*, a gene implicated in type-2 diabetes pathology and VSMC contraction^40^, is present near the enhancer-associated lncRNA *lnc-Ang383* (Fig. 5b) and *Ramp3* was induced 3–6 h post AngII treatment (Fig. 5g). To determine whether the AngII-regulated enhancer and overlapping *lnc-Ang383* are involved in regulating *Ramp3* expression, we used loss-of-function approaches. We first used specific Dicer-substrate siRNAs (DsiRNAs) to knockdown *lnc-Ang383* in RVSMCs. Interestingly, *lnc-Ang383* DsiRNAs significantly attenuated the AngII-induced expression of not only *lnc-Ang383* but also its nearby gene *Ramp3* compared to non-targeting Control DsiRNA ± AngII treatment (Fig. 5h). Due to the relative inefficiency of siRNAs to target lncRNAs, we next performed CRISPR/Cas9-mediated deletion of the enhancer region overlapping *lnc-Ang383* (Supplementary Fig. 9a). Using droplet digital PCR (ddPCR), we determined the deletion allele frequency to be 48.5%. However, the proportion of homozygous lncRNA (KO) cells in the pooled population is presumably much lower as we are comparing PCR products from pooled genomic DNA of heterogeneous population of cells (see Methods). Notwithstanding, results clearly showed that AngII-induced *Ramp3* as well as *lnc-Ang383* expression was significantly attenuated upon enhancer deletion (Fig. 5i, j). Notably, deletion of this enhancer also significantly attenuated AngII-induced expression of *Ccl2*, *Serpine1*, and *Il6* in RVSMCs (Fig. 5k-m), implying that *lnc-Ang383* might play an important role in shaping the AngII-induced inflammatory/fibrotic gene network in VSMC through its overlapping enhancer. Moreover, we also found that an AngII-induced enhancer overlaps *lnc-Ang362* which is induced by AngII and plays a role in VSMC growth^11^ (Supplementary Fig. 10a, b). Altogether these data suggest that key lncRNAs and overlapping enhancers may play important functional roles in the expression of AngII-induced nearby genes.

### Identification of AngII-regulated SEs

**Fig. 6:**
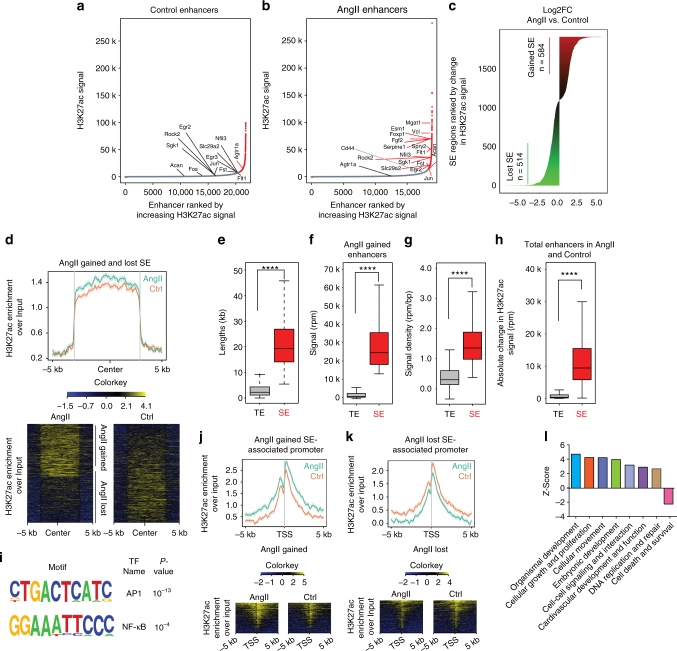
Characterization of AngII-induced SEs in VSMCs. **a**, **b** Ranked plots of enhancers in Control (**a**) or AngII-treated (**b**) RVSMCs ranked by increasing H3K27ac signal. Enhancers are defined as overlapping regions of H3K27ac and H3K4me1 and with differential enrichment of H3K27ac. SEs are colored red and TEs are colored in gray. The SE and TE-associated genes are indicated in the figure. **c** SEs in Control and AngII-treated RVSMCs are shown ranked by log2 fold change in H3K27ac signal (AngII vs. control). The change in H3K27ac levels at SEs is indicated by change in color intensity (green to red). **d** Average profile and heatmaps of H3K27ac enrichment over AngII gained and lost SEs. **e**–**h** Boxplots show median enhancer length (kb) (**e**), signal (rpm) (**f**), and density (rpm/bp) (**g**) in AngII gained TEs and SEs. **h** Boxplot shows the absolute change in H3K27ac signal in response to AngII treatment measured at all SEs in Control and AngII-treated RVSMCs. For **e**–**h** ****P < 0.0001, using unpaired two-tailed *t*-test. **i** TF binding motifs enriched in AngII gained SEs. De novo motif discovery identified AP1 motif to be enriched (*P* < 10^−10^, binomial distribution) while NF-κB motif was enriched in known motifs (*q* < 0.005, Benjamini multiple hypothesis testing). **j** Average profile and heatmaps of H3K27ac enrichment over promoters of AngII gained and **k** AngII lost SE-associated genes. **l** IPA Pathway analysis of genes associated with gained SEs

Enhancers with multiple/broad H3K27ac signals are designated as SEs and may determine cell identity in normal and disease states^20,22^. SEs can also be marked by enrichment of the epigenetic reader protein, BRD4^22,23^. Since differential regulation of SEs can yield novel information about genes involved in VSMC dysfunction, we identified AngII-regulated SEs in RVSMCs using the ROSE algorithm^22,41^ that stitches enhancers present within 12.5 kb of each other and ranks them according to peak score. We used H3K27ac and H3Kme1 co-occupied regions (enhancers) identified in Control and AngII conditions that were differentially enriched for H3K27ac to call H3K27ac-specific SEs (Fig. 6a, b). Additionally, we performed BRD4 ChIP-seq on Control and AngII-treated RVSMCs to identify BRD4-specific SEs. We identified 660 and 1245 H3K27ac-specific SEs and 1245 and 571 BRD4-specific SEs in AngII-treated and Control VSMCs, respectively, which are lower than typical enhancers (TEs) (Supplementary Fig. 11a–d). Whereas 504 SEs combined were common in both H3K27ac and BRD4 data sets in control and AngII conditions (Supplementary Fig. 11e, f). We used the SEs identified in the H3K27ac data set for all further comparisons. AngII treatment led to widespread changes in H3K27ac occupancy at several SEs (Fig. 6c). Differential enrichment analysis revealed that 584 SEs were gained upon AngII treatment in contrast to 514 lost SEs (Fig. 6c, d). The average profile of H3K27ac enrichment over all differentially regulated SEs was higher in the AngII-treated condition (Fig. 6d). AngII gained SEs (Fig. 6e–g) as well as lost SEs (Supplementary Fig. 12a–c) were significantly larger in length and showed higher H3K27ac signal and signal density relative to TEs. AngII induced a greater change in H3K27ac signal at SEs as compared to TEs which was even more robust in the AngII gained SEs (Fig. 6h and Supplementary Fig. 12d). BRD4 enrichment was largely consistent with H3K27ac enrichment over gained and lost SE regions (Supplementary Fig. 12e). Moreover, AngII treatment induced co-ordinate changes in H3K27ac and BRD4 enrichment over AngII gained and lost SEs (Supplementary Fig. 13a–d). Motif discovery analysis and known TF binding site enrichment analysis on the upregulated SEs showed significant enrichment of binding sites for AP1 and NF-κB (Fig. 6i). Genes proximal to the AngII gained SEs showed increased enrichment of H3K27ac and BRD4 at their promoters following AngII treatment (Fig. 6j and Supplementary Fig. 14a). Conversely, genes proximal to AngII lost SEs exhibited decreased enrichment of H3K27ac and BRD4 at their promoters (Fig. 6k and Supplementary Fig. 14b). IPA analysis of AngII-regulated SE (Fig. 6l) and TE-associated (Supplementary Fig. 15) genes revealed enrichment of functions related to organismal development, cellular growth and proliferation, and cardiovascular development and function, suggesting a pivotal role of the SE-associated genes in CVDs, and that SE-associated genes may be significant biomarkers for VSMC identity and dysfunction.

**Fig. 7:**
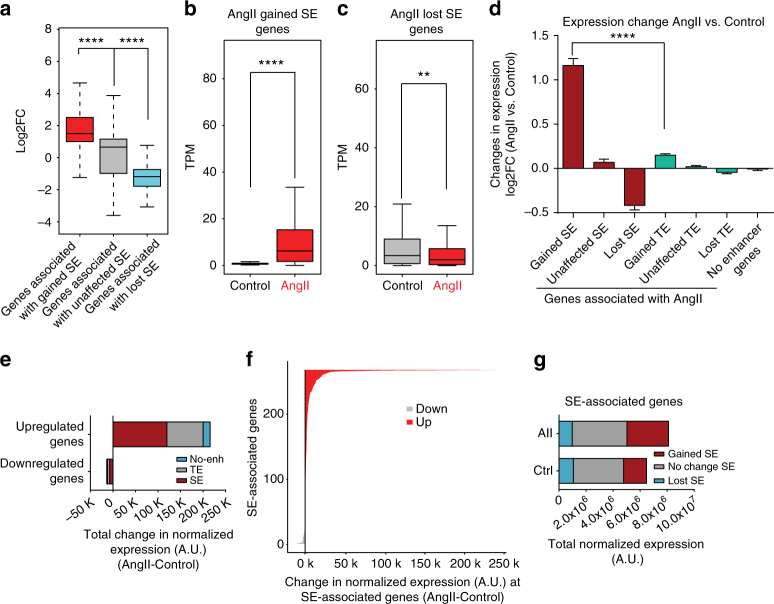
SEs regulate proximal gene expression in response to AngII. **a** Boxplot shows average log2 fold change in mRNA expression at genes associated with AngII gained, AngII lost, or unaffected SEs. **b** Boxplot shows TPM of genes associated with SEs that were gained or **c** lost in response to AngII treatment. **P < 0.01; ****P < 0.0001 (for **a**–**c**), using unpaired two-tailed *t*-test. **d** Bar plot shows average log2 fold change in mRNA expression at genes associated with AngII gained, AngII lost, or unchanged SE, TEs and genes associated with no enhancers. Error bars represent SEM. ****P < 0.0001, using one-way ANOVA, Tukey’s multiple comparisons test. **e** Stacked bar graph shows the cumulative change in gene expression after AngII treatment at upregulated or downregulated genes (−1 > log2FC > 1). Genes are further categorized based on whether they are associated with SEs, TEs or are not associated with any enhancers. **f** Plot shows the absolute change in normalized expression (A.U.) post AngII treatment for all SE-associated genes. Genes are sorted by absolute change in expression between AngII-treated and Control cells with red and gray depicting upregulated and downregulated genes, respectively. **g** Stacked bar graph shows the cumulative expression of genes associated with AngII gained, lost or unchanged SEs in Ctrl and AngII-treated RVSMCs. AngII treatment induced a 25% increase in total normalized expression of SE-associated genes and 21% of this increase was contributed by AngII gained SE-associated genes

Furthermore, integration with RNA-seq data from AngII-treated RVSMCs^11^ showed that genes associated with gained SEs had significantly higher fold changes in expression compared to genes associated with lost or unaffected SEs (Fig. 7a). This trend was also visible when transcripts per million reads (TPM) were compared in Control and AngII conditions for gained and lost SE-associated genes (Fig. 7b, c). Moreover, the expression changes for SE-associated genes were more robust than TE-associated genes (Fig. 7d). Overall, the SE-associated genes contributed to more than 50% of the total increase in upregulated gene expression (Fig. 7e) and, among the SE-associated genes, more were upregulated than downregulated (Fig. 7f). Moreover, AngII gained SE-associated genes contribute to about 21% of the 25% increase in the mRNA levels of SE-associated genes upon AngII treatment (Fig. 7g).

### JQ1 attenuates AngII-induced SE-associated gene expression

**Fig. 8:**
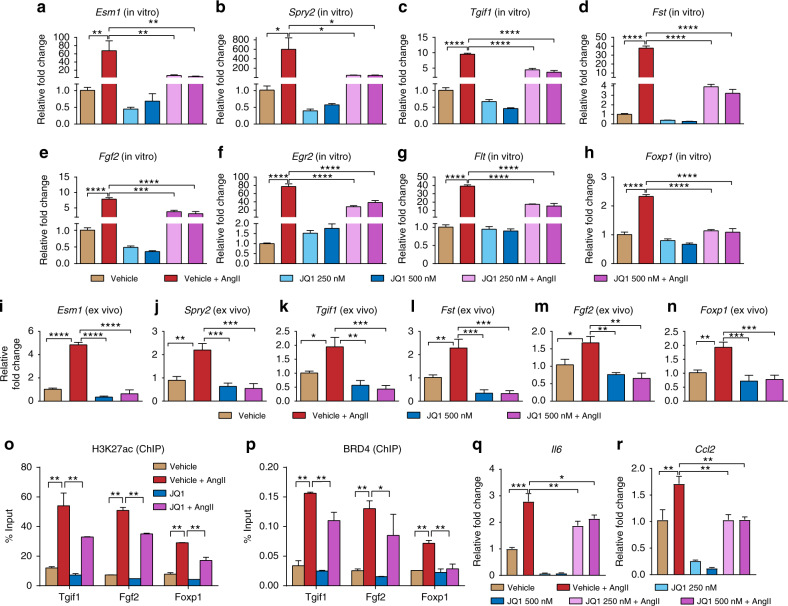
JQ1 attenuates SE formation and SE-associated gene expression. **a**–**h** and **q**, **r** Bars represent the expression of SE-associated genes or inflammatory genes measured by RT-qPCR in RVSMCs pre-treated with vehicle or BRD4 inhibitor JQ1 (250 and 500 nM) and then treated in vitro with AngII as indicated. **i**–**n** Bars represent the expression of indicated SE-associated genes in rat aortas pretreated ex vivo with vehicle or JQ1 (500 nM) followed by AngII treatment. Gene expression was normalized with *Ppia* and represented as relative fold change with respect to vehicle. Mean + SEM, *n* = 3. For **i**–**n** (*n* = 4 aortas). *P < 0.05; **P < 0.01; ***P < 0.001; ****P < 0.0001, using one-way ANOVA, Tukey’s multiple comparisons test. **o**, **p** ChIP assays. Bars represent H3K27ac (**o**) and BRD4 (**p**) ChIP enrichment on the indicated SE regions in RVSMCs untreated or treated with either AngII, JQ1, or both in vitro. Data are represented as Mean + SEM, *n* = 3. *P < 0.05; **P < 0.01, using one-way ANOVA, Tukey’s multiple comparisons test

To further confirm the role of SEs, we used JQ1, a well-characterized BET bromodomain inhibitor. JQ1 is a competitive inhibitor of acetylated lysine, binding to the bromodomains of BRD3 (*K*_d_ ∼59 nM), BRD4 (*K*_d_ ∼49 nM), and BRD2 (*K*_d_ ∼128 nM)^42^ that can disrupt functions of SEs in cancer, inflammation and atherogenesis through BRD4 inhibition^23,42,43^. Since the expression and AngII regulation of these BET family proteins is not known in RVSMCs, we first checked AngII-induced expression of *Brd2*, *Brd3*, and *Brd4*. Results showed that *Brd4* is slightly, but significantly induced by AngII treatment, but not *Brd2* or *Brd3* (Supplementary Fig. 16). The effect of JQ1 on the expression of upregulated SE-associated genes was determined by pre-treating RVSMCs with JQ1 (250 and 500 nM, 2 h) followed by AngII treatment for 3 h. JQ1 markedly attenuated AngII-induced expression of several SE-associated genes (Fig. 8a–h). These JQ1 effects on candidate genes were also reproduced in ex vivo treated rat aortas (Fig. 8i–n). ChIP assays with H3K27ac and BRD4 antibodies further verified attenuation of AngII-induced SE formation at candidate regions in RVSMCs treated with JQ1 + AngII vs. AngII alone (Fig. 8o, p). Moreover, JQ1 attenuated AngII-induced expression of pro-inflammatory genes *Il6* and *Ccl2* in RVSMCs (Fig. 8q, r). Furthermore, JQ1 also attenuated AngII-induced expression of *lnc-Ang383* and its nearby gene *Ramp3* (Supplementary Fig. 17). Together, these results clearly illustrate the important role of SEs in AngII-induced gene expression in VSMCs.

### SE deletion blocks AngII induction of its nearest gene

**Fig. 9:**
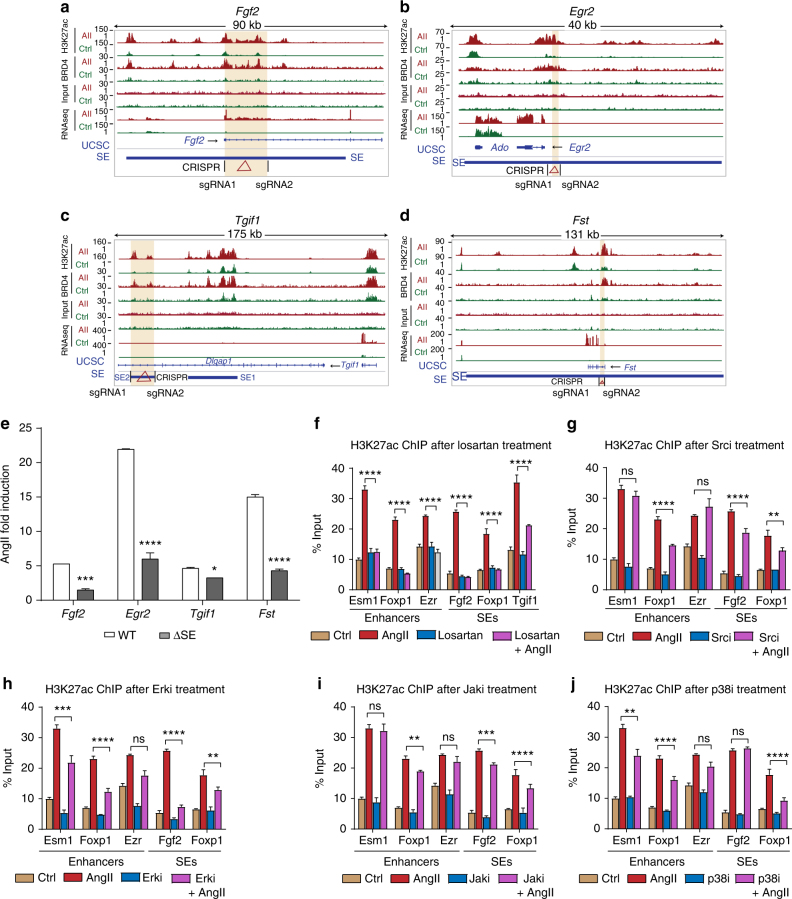
SE deletion blocks the expression of corresponding nearby genes. **a**–**d** Genome browser tracks showing the relative position of the indicated SEs (highlighted in orange) with respect to nearby indicated gene and the position of the sgRNAs (denoted by black lines) designed to delete the SE using CRISPR-Cas9 editing; **a** *Fgf2*, **b** *Egr2*, **c** *Tgif1*, **d** *Fst*. **e** Bar graphs show the AngII-induced expression of indicated genes in WT and SE deleted (∆SE) RVSMCs mutants. Gene expression was normalized to *Ppia* and expressed as AngII response vs. respective Controls. Mean + SEM, *n* = 3. *P < 0.05; ***P < 0.001; ****P < 0.0001, using unpaired two-tailed *t*-test. **f**–**j** Signaling mechanisms involved in AngII-induced regulation of enhancers/SEs in VSMCs. Bars represent H3K27ac enrichment (ChIP assays) on the indicated enhancer and SE regions in control (Ctrl) or AngII-stimulated RVSMCs pretreated with or without inhibitors (**i**) of AT_1_R (Losartan), Src (PP1), Erk1/2 (U0126), Jak (Inhibitor 1), and p38MAPK (SB202190). Data are represented as Mean + SEM, *n* = 3. **P < 0.01; ***P < 0.001; ****P < 0.0001; ns non-significant, using one-way ANOVA, Tukey’s multiple comparisons test

To further determine the functional involvement of SEs on the expression of AngII-regulated genes, we carried out CRISPR-Cas9-mediated genome editing to delete SE regions associated with candidate AngII-induced genes such as *Fgf2*, *Egr2*, *Tjpg1*, and *Fst*. Target-specific guide RNAs (sgRNAs) targeting a part of the SEs (based on location of higher H3K27ac or BRD4 enrichment) identified in both H3K27ac and BRD4 data sets (*Fgf2* and *TJPEG1*); and SEs specific to H3K27ac (*Egr2*) or BRD4 (*Fst*) were designed (Fig. 9a–d). The SE deletions were confirmed by genotyping (Supplementary Fig. 9b–e). Next, AngII-induced expression of SE-associated genes in SE deleted cells was assessed by RT-qPCR. Results showed that deletion of SEs significantly decreased AngII-induced expression of *Fgf2*, *Egr2*, *Tgif1*, and *Fst* (Fig. 9e). It is possible the effects observed on nearby gene expression after SE KO could be underestimated due to the relative inefficiency of CRISPR-Cas9-mediated KO using two sgRNAs in transfected primary RVSMCs and lower abundance of the homozygous KO population in the pooled cells analyzed.

To determine whether SE deletion has any effect on the second and third nearby genes of the tested SEs, we performed RT-qPCR analysis on candidate SE deleted cells after AngII treatment. Results clearly showed that deletion of the indicated SEs does not affect the expression of respective second and third nearby genes, i.e., *Nudt6* and *Spata5* (for Fgf2), *Ado* and *Nrbf2* (for Egr2), *Dlgap1* and *Myom1* (for Tgif1), and *Ndufs4* and *Mocs2* (for Fst) (Supplementary Fig. 18a–d). In addition, comparison of expression of genes mapped to differentially regulated SEs and TEs showed that changes in expression of the first nearest genes correlate well with changes in SE activity compared to the second and third (Supplementary Fig. 18e). Moreover, genes mapped to TEs showed relatively lower correlation with changes in expression and H3K27ac enrichment at TEs (Supplementary Fig. 18f). Together our data strongly support the involvement of AngII-induced SEs in the regulation of AngII-induced genes involved in VSMC dysfunction.

### Multiple signaling events mediate enhancer formation

In order to investigate the signaling events involved in AngII-mediated enhancer and SE formation, we examined the effects of inhibitors of AT_1_R and various signaling pathways known to be downstream of AT_1_R, such as Src, JAK, ERK, and p38 kinases^2,5,7,8,44^. We performed H3K27ac ChIP on three enhancers and two-three SEs with cells pre-treated with these inhibitors (1 h) followed by AngII treatment (3 h). Results showed that AT_1_R antagonist, losartan, completely blocked the induction of all the tested enhancers and SEs, confirming the specific role of AngII signaling via AT_1_R (Fig. 9f). Moreover, inhibitors of kinases downstream of AT_1_R (including Src, JAK, ERK, and p38), depicted distinct inhibitory effects, with individual enhancers/SEs affected by different signaling pathway inhibitors (Fig. 9g–j). Hence, there seems to be an integration of signals from multiple pathways downstream of the AngII receptor in shaping the enhancer and SE landscape.

### JQ1 ameliorates AngII-induced hypertension in mice

**Fig. 10:**
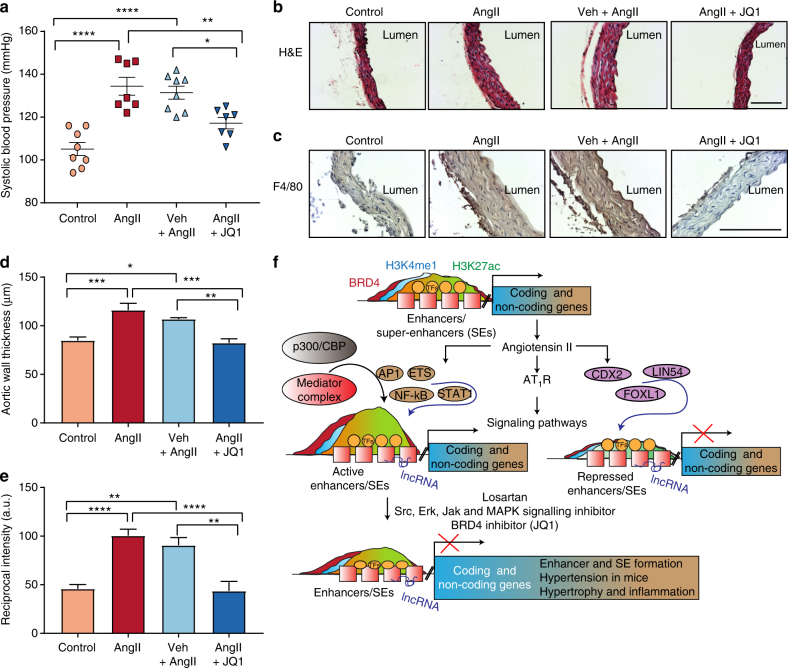
JQ1 ameliorates hypertension, hypertrophy and inflammation in AngII-infused mice. C57BL/6 mice were infused with PBS (Control) or AngII and AngII-infused mice were also treated with vehicle (Veh + AngII) or JQ1 (AngII + JQ1) for 4 weeks. **a** Scatter plot showing systolic blood pressure (mmHg) of mice from indicated groups. Data are represented as Mean + SEM, *n* = 7 for AngII and AngII + JQ1 groups, *n* = 8 for Control (PBS) and Veh + AngII groups. *P < 0.05; **P < 0.01; ****P < 0.0001, using one-way ANOVA, Tukey’s multiple comparisons test. **b** Representative images of H&E staining in mouse aortas from indicated groups. 10× magnification, scale bar 150 µm. **c** Representative images of F4/80 staining in mouse aortas from indicated groups. 20× magnification, scale bar 150 µm. **d**, **e** Quantification of **b** and **c** (as described in Methods). Data are represented as Mean + SEM, *P < 0.05; **P < 0.01; ***P < 0.001; ****P < 0.0001, using one-way ANOVA, Tukey’s multiple comparisons test. **f** Scheme depicting AngII-induced epigenetic changes at enhancers/SEs leading to remodeling and altered gene regulation. Unstimulated VSMCs maintain basal levels of gene expression that are associated with enhancers/SEs with minimum or moderate H3K4me1, H3K27ac, and BRD4 signals. These enhancers/SEs may be occupied by a limited number of TFs. AngII stimulation and subsequent downstream signaling possibly leads to increased recruitment of TFs such as AP1, ETS, STAT1 and NF-κB at their respective binding sites on the enhancers/SEs that further recruit BRD4, the mediator complex, and p300/CBP leading to increased H3K27ac enrichment and SE formation thereby enhancing the transcription of nearby genes. Moreover, AngII stimulation could also recruit other TFs such as CDX2, FOXL1 and LIN54 and through an unknown mechanism may lead to a loss of H3K27ac enrichment on the enhancer to downregulate the associated genes. Enhancer-associated lncRNAs might be intimately linked to both these processes and may play a crucial role in AngII-induced gene expression in VSMCs. Blocking AngII signaling by the AT_1_R blocker losartan or inhibitors of downstream signaling pathways attenuates AngII-induced enhancer/SE formation. Moreover, blocking SEs using a BRD4 inhibitor JQ1 ameliorates AngII-induced hypertension in mice

To determine the effect of JQ1 on AngII-induced hypertension in vivo, we performed AngII infusion in mice with or without JQ1 (50 mg kg^−1^) administration (see Methods). Results clearly showed that AngII infusion significantly increased systolic blood pressure compared to the Control group. Notably, JQ1 injection significantly attenuated AngII-induced increases in systolic blood pressure compared to AngII alone or Veh + AngII (Fig. 10a). Moreover, JQ1 attenuated AngII-induced medial hypertrophy and inflammation (F4/80 staining) compared to AngII alone or Veh + AngII (Fig. 10b–e). These data clearly suggest that AngII-induced hypertension, a key CVD phenotype, is ameliorated after BET inhibition by JQ1 in our animal model system.

## Discussion

Phenotypic transition of VSMCs from a contractile to synthetic state, induced by GFs like AngII, is implicated in CVDs such as hypertension, atherosclerosis, and restenosis. To determine epigenetic mechanisms involved in these processes, we profiled for the first time the enhancer and SE repertoire in control and AngII-stimulated RVSMCs, which yielded a rich resource of epigenomic data of VSMC distal regulatory elements (enhancers/SEs) controlling the expression of critical genes and processes involved in CVDs. Our data suggest that unstimulated VSMCs maintain basal levels of gene transcription associated with enhancers and SEs having minimum or moderate enrichment of H3K4me1, H3K27ac, and mediator protein BRD4. In this scenario, the enhancers/SEs are likely co-occupied by a limited number of lineage or cell type-specific TFs^45,46^. Upon AngII stimulation, a cascade of downstream signaling events involving various kinases recruit key signal-dependent TFs, such as AP1, ETS1, NF-κB, and STAT, to not only proximal promoters, but also distal enhancers/SEs that further recruit HATs and mediator complexes, including BRD4. This enhances H3K27ac enrichment to augment the transcription of AngII-responsive genes. Furthermore, we found that certain AngII-responsive lncRNAs overlap AngII-regulated enhancers and appear to be associated with corresponding enhancer activity and expression of nearby genes in VSMCs (Fig. 10f). AngII also recruits other TFs such as CDX2, LIN54, and FOXL1 along with loss of H3K27ac at enhancers/SEs by as-yet unknown mechanisms, thereby downregulating other genes.

TFs work together in tandem at enhancers and SEs in a signal-dependent manner to regulate gene expression. Numerous key TFs acting at promoters have been shown to mediate VSMC growth and inflammatory responses^2,8,27–30,44,47^. Our de novo motif discovery analyses for *cis*-elements within AngII-regulated enhancers and SEs depicted enrichment for AP1, ETS, NF-κB, and STAT sites suggest that these regulatory factors/elements acting at enhancers most likely contribute to cell type-specific regulation of AngII functions in VSMCs. Interestingly, AngII treatment also increased H3K27ac at enhancers near AP1 (*c-jun*) and *Ets2* (Fig. 1), suggesting that AP1 and ETS family members function in a regulatory feedback loop, by not only regulating their own expression but also controlling various AngII-regulated enhancers in VSMCs. However, not all the enhancer/SEs are dependent on AP1 and ETS occupancy, suggesting a role for other TFs such as STAT and NF-κB (acting alone or in conjunction with AP1) in the formation of enhancers/SEs in VSMC. The binding of these TFs is coupled to the dynamic remodeling of the chromatin around enhancers (indicated by changes in H3K27ac), ultimately driving AngII-regulated gene expression patterns in VSMCs. Previous studies of classic AngII-responsive genes in VSMCs and other cells have demonstrated clear roles for promoter AP1 and other TFs^5,7,8,27–30,48^. However, in the current study we show for the first time a new functional role for these TF sites, particularly AP1, in distal enhancers in the AngII response. *Ets1* was also induced by AngII in RVSMCs, in agreement with previous studies^27,49^. *Ets1* expression promotes VSMC proliferation by inducing platelet-derived growth factor (PDGF) and PDGF receptor. Ets1 can promote phenotypic switching of VSMC associated with increased proliferation and decreased expression of VSMC-specific marker genes, including SM α-actin, SM MHC, and SM22α^50^. Our studies now suggest ETS1 can contribute, at least in part to AngII-induced enhancer formation. GSEA analysis of upregulated enhancer-associated genes also revealed that SRF targets were the most highly upregulated upon AngII treatment (Fig. 4e). This is noteworthy because SRF is highly implicated in VSMC differentiation and immediate early gene expression^51^. However, as we did not observe SRF binding motifs in AngII-upregulated enhancers, signal-dependent TFs like AP1 and ETS might function upstream of SRF (a lineage-specific TF) during de novo enhancer formation. This is consistent with the fact that we used cultured VSMCs (±AngII) in the synthetic phenotype. Studies in endothelial cells demonstrated that NF-κB activation by the pro-inflammatory cytokine TNF-α alters the enhancer landscape and contributes to SE formation^23^. However, TFs involved in enhancer/SE regulation in VSMCs have not been previously examined. Our data demonstrates that AP1 binding motifs are the most highly enriched in AngII-regulated VSMC enhancers/SEs, suggesting a critical role for AP1 in enhancer/SE formation and associated gene expression in VSMCs. Thus, our results illustrate cell-specific and stimulus-specific differences in TFs regulating enhancer activity.

We also validated the AngII-induced alterations in H3K27ac at candidate enhancers and their nearby AngII-regulated genes, such as *Esm1*, *Spry2*, and *Agtr1a* in vitro, ex vivo and in vivo (Figs. 2, 3). These genes are involved in VSMC growth, proliferation, inflammation, and hypertension, suggesting a key role for enhancers in VSMC dysfunction^32–34^. Furthermore, some of these in vivo changes were ameliorated in AngII-infused AT_1_RKO mice, supporting the involvement of AT_1_R signaling in the modulation of enhancers, data also supported by the inhibitory effects of the AT_1_R antagonist, losartan, as well as antagonists of downstream signaling pathways (Fig. 10f).

Evidence shows that the noncoding portion of the genome contains *cis*-regulatory sequences and associated noncoding RNAs with crucial regulatory functions within networks that dictate cardiac development and disease^52^. Our data suggest that AngII-induced lncRNAs associated with enhancer/SE regions might functionally contribute to the expression of AngII-induced nearby genes. This scenario is consistent with emerging roles of enhancer-associated lncRNAs in modulating the expression of nearby protein-coding genes^38,39^. Potential roles for AngII-regulated enhancer lncRNAs include modulation of VSMC growth and inflammatory phenotype, as supported by our data showing inhibition of inflammatory gene expression by deletion of the *lnc-Ang383*-associated enhancer (Fig. 5). Further studies will be needed to determine the mechanisms involved and how these unique layers of the genome/epigenome/transcriptome (enhancers, SEs, and lncRNAs) co-operate during AngII-mediated gene expression.

Brown et.al. showed that the BET (BRD4) inhibitor JQ1 disrupts SEs associated with inflammatory gene expression, and suppresses atherogenesis in hypercholesterolemic mice by attenuating inflammation and endothelial cell activation^23^. JQ1 was also used to demonstrate BET-regulated transcriptional programs during cardiomyocyte hypertrophy^53^. From their data, it appears that the SEs in cardiomyocytes and VSMC can regulate genes with potentially similar functions (cell growth, hypertrophy, and inflammation), highlighting the critical role of SEs in regulating CVD phenotypes in these two important AngII-responsive cells. However, as TEs and SEs are cell-specific, they can yield important clues to the cell-specific functions of AngII and other stimuli in distinct pathophysiological settings (hypertension vs. heart failure). Similarly, BET inhibition in endothelial dysfunction and atherosclerosis shows some similarities, but also distinct differences from our data. Notably, unlike ours, these reports did not examine lncRNA targets which could shed new light on the mechanisms of actions of BET inhibitors. Our data show that JQ1 significantly attenuates AngII-regulated SE formation (H3K27ac and BRD4 occupancy) (Fig. 8o, p), and also AngII-induced expression of SE-associated genes both in vitro and ex vivo. We further demonstrated similar outcomes after CRISPR-Cas9-mediated deletion of relevant SE regions (Fig. 9). Moreover, we observed that JQ1 attenuates AngII-induced hypertension, medial hypertrophy, and inflammation in vivo in mice. Together, these data suggest an important role of BET bromodomains in SE formation in RVSMCs, and in AngII-induced gene regulation and related CVDs.

Emerging data show that several disease-associated SNPs are enriched in enhancers and SEs^21,54^. Using liftOver tool^55^ we identified human genomic regions orthologous to AngII-regulated H3K27ac enhancers in rat. Notably, these regions in the human genome harbor known GWAS/SNPs for AngII-related CVD disease/traits, including abdominal aortic aneurysms (AAAs), atrioventricular conduction, coronary heart disease, diastolic blood pressure, glucose homeostasis traits, inflammatory skin disease, obesity-related traits, pulmonary function decline, systolic blood pressure, and Type 1/2 diabetes (Supplementary Table 2). Future studies with VSMC from subjects enrolled in appropriate clinical trials are needed to verify functional connections between these VSMC enhancers/SEs, disease-related SNPs and CVDs.

Together, our findings provide a new perspective on the epigenetic mechanisms underlying AngII actions and VSMC dysfunction, including previously unrecognized roles of enhancers and SEs in VSMC, and their potential connections to CVDs, data which could be exploited for drug discovery. BET inhibitors like JQ1, and related small molecules that interfere with SEs, have been shown to be effective in animal models of atherosclerosis, AAA^56^, inflammatory renal disease^57^, pulmonary hypertension^58^, carotid intimal hyperplasia^59^ and heart failure^60^, and in clinical trials for certain cancers^61^. Our data showing that JQ1 inhibits AngII-induced hypertension further underscores the promise of such epigenetic modifiers for the treatment of human CVDs. Thus, AngII-regulated enhancers/SEs, and their associated lncRNAs could represent novel targets for the development of better drugs for hypertension, atherosclerosis, and renal diseases, especially for patients who do not respond well to currently available AngII receptor blockers and ACE inhibitors^4^.

## Methods

### Isolation of RVSMCs and treatment with AngII

All animal experiments were performed in accordance with protocols approved by the Institutional Animal Care and Use Committee. RVSMCs were isolated from thoracic aortas of 12-week-old male Sprague-Dawley rats (Charles River, San Diego, CA) by enzymatic digestion^10,11^. Briefly, aortas were excised and transferred to a plate containing sterile PBS. Connective tissues were removed using fine forceps followed by collagenase (Sigma, St. Louis, MO) and elastase (Sigma) digestion (2 mg ml^−1^ each) for 10–20 min at 37 °C to loosen the adventitial layer. This layer was removed with forceps and the aortas subjected to second digestion for 1–2 h at 37 °C to loosen the smooth muscle cells, then dispersed using a 1 ml pipette and passed through a cell strainer. The suspension was mixed with M199 medium (Corning Cellgro, Manassas, VA) and centrifuged at 3000 rpm for 5 min at 4 °C to collect the cells. The cell pellet was resuspended in M199 medium supplemented with 20% FBS and transferred to dishes to adhere at 37 °C in 5% CO_2_. RVSMCs obtained thereby were cultured in M199 medium supplemented with 10% FBS, 1% penicillin/streptomycin, and 2.5 µg ml^−1^ plasmocin. Prior to AngII treatment, RVSMCs were serum depleted in M199 medium containing 0.2% BSA for 48 h and then stimulated with 100 nM AngII (Bachem, Torrance, CA) or left untreated (Control) for indicated time periods.

### Ex vivo cultures of rat aortas

Aortas from 12-week-old male Sprague-Dawley rats were excised under aseptic conditions and adventitia carefully stripped followed by extensive rinsing in PBS. Each aorta was divided into four pieces, and were either incubated in serum-free M199 medium without AngII (control) or with AngII (100 nM) for different time points (1, 3, and 6 h). Subsequently, the aortic specimens were homogenized in QIAzol (Qiagen, Valencia, CA), followed by RNA isolation and gene expression analysis or processed for ChIP assays.

### Inhibition of BRD4 using JQ1

RVSMCs and aortic tissues were pretreated with the BET bromodomain/BRD4 inhibitor JQ1 (250 or 500 nM) or vehicle DMSO for 2 h prior to treatment ± AngII (100 nM, 3 h). Then samples were lysed in QIAzol to extract total RNA for candidate gene expression analysis by RT-qPCR or processed for ChIP assays with specific antibodies.

### Treatment with inhibitors of AT_1_R and signaling pathways

RVSMCs were pretreated with various signaling pathway inhibitors; Src (PP1) (10 µM) (Calbiochem, Billerica, MA), Phospho-p42/p44 MAPK or Erk1/2 (U0126) (10 µM) (Cell Signaling Technology, Danvers, MA), JAK (Inhibitor I) (10 µM) (Calbiochem), p38 MAP kinase (SB202190) (5 µM) (Cell Signaling Technology), and AT_1_R antagonist losartan (10 µM) (Merck, Whitehouse Station, NJ) or vehicle DMSO for 1 h prior to treatment ± AngII (100 nM, 3 h). The cells were then processed for ChIP assays with specific antibodies.

### AngII infusion of mice (in vivo experiments)

Male C57BL/6 mice (stock number 000664) and AT_1_R knockout (AT_1_RKO) (stock number 002682) mice (Jackson Laboratory, Bar Harbor, ME) were implanted with osmotic minipumps (Alzet, model 2002, Cupertino, CA) to infuse AngII or vehicle PBS as described previously^10,62^. Briefly, an incision was made in the mid-scapular region, and an osmotic minipump filled with AngII or vehicle (PBS) was implanted subcutaneously (12 mice/group) and incision was closed with surgical glue. Using this protocol, AngII was delivered at a concentration of 1.0 μg kg^−1^ min^−1^ for 28 days. At the end of the experiment, animals were euthanized, aortas were collected, and adventitia removed carefully as described before^10^. Subsequently aortic tissues were processed for gene expression analysis by RT-qPCR and for ChIP assays.

### AngII infusion of mice and administration of JQ1

Male C57BL/6-WT mice were divided into four groups; PBS, AngII, vehicle + AngII, and AngII + JQ1 (eight mice per Control (PBS) and vehicle + AngII groups and seven each per remaining two groups). Osmotic mini pumps containing AngII or PBS were implanted in mice as described before^10^. 2 days post-implantation, JQ1 (Shanghai Chempartner Co., Ltd/China Gateway Life Science (Holdings) Limited, Shanghai, China) was administered intraperitoneally in AngII + JQ1 group at 50 mg kg^−1^, a dose reported previously^23^. Vehicle + AngII group was injected with vehicle alone (DMSO). JQ1 or its vehicle were administered once daily until the end of the experiment (up to 28 days). During the 4th week of the experiment blood pressure was measured using the tail cuff method (Visitech, Apex, NC).

### Immunohistochemistry

Formalin-fixed, paraffin-embedded mouse aortas were cut into 5 µm thick sections and mounted onto positively charged slides. For Hematoxylin and Eosin (H&E) staining, deparaffinized slides were cleared in absolute alcohol, rinsed in distilled water and stained using Mayer’s Hematoxylin, followed by bluing in tap water. Slides were then dipped in 80% alcohol and stained with (1%) eosin. Finally, samples were gradually dehydrated in increasing strength of alcohol, cleared in xylene and mounted with Cytoseal 60 mounting media. Immunohistochemical staining of F4/80 was performed on deparaffinized aortic sections. Samples were quenched in 3% hydrogen peroxide and pretreated to promote antigen retrieval by steam in DIVA buffer (pH 6.2) solution for 20 min. After antigen retrieval, slides were blocked in 10% normal rabbit serum for 10 min, incubated in F4/80 monoclonal primary antibody (1/50) (eBioscience, Waltham, MA) for 60 min, washed in PBS and incubated in biotinylated rabbit anti-rat IgG secondary antibody (1/200) (Vector Laboratories Inc., Burlingame, CA). Subsequently, PBS washed samples were labeled with ABC Elite streptavidin-peroxidase (Vector Laboratories Inc.), incubated with the chromogen diaminobenzidine tetrahydrochloride (DAB), and mounted after counterstaining with hematoxylin. All H&E and F4/80 images were captured at 10× and 20× magnification, respectively, using Zeiss observer microscope. Fiji (ImageJ) software were used to quantify aortic wall thickness from the H&E images, and brown stain intensity from the DAB stained images. DAB stained bright field images were color deconvoluted using Fiji software and only the brown colored images were traced to exclude the adventitia before measuring the mean gray value. Reciprocal intensities were calculated by subtracting mean intensity values from the maximum intensity value (255) to obtain a positive correlation with the chromogen stain. For H&E quantification and statistics, aortas from at least 4 mice per group and at least 12 aortic sections per group were analyzed (Control, *n* = 7 mice; AngII, *n* = 6 mice; Veh + AngII, *n* = 4 mice; AngII + JQ1, *n* = 6 mice). For F4/80 quantification and statistics, aortas from at least four mice and at least four aortic sections per study group were analyzed (Control, *n* = 5 mice; AngII, *n* = 6 mice; Veh + AngII, *n* = 4 mice; AngII + JQ1, *n* = 4 mice). Investigators analyzing the histopathological data derived from animal models were not masked to the treatment of the animals.

### RT-qPCRs of enhancer-associated RNAs (coding/non-coding)

To assess levels of enhancer-associated transcription (coding and non-coding), total RNA was isolated from control and AngII-treated RVSMCs and aortic tissues using RNeasy mini kit (Qiagen). Aortas were homogenized using green RINO beads (Next Advance, Averill Park, NY) in a Bullet Blender (Next Advance) before proceeding for RNA isolation. To remove genomic DNA contamination, RNA samples were digested on-column with RNase-Free DNase I (Qiagen). For coding genes, cDNA was synthesized from 1 µg of total RNA using the Prime Script^TM^ RT Master Mix (Takara, Mountain View, CA). Whereas for non-coding transcripts, cDNA was synthesized using the QuantiTect Reverse Transcription Kit (Qiagen), which includes an additional DNase digestion step to eliminate genomic DNA contamination. Gene expression was analyzed by quantitative PCR (qPCR) using SYBR Green reagent (Applied Biosystems, Foster City, CA) with gene-specific primers (Supplementary Table 1) in 20 µl reactions in triplicate on 7500 Fast Real-Time PCR system (Applied Biosystems). Relative gene expression levels between control and treated groups were determined using 2^−ΔΔCt^ method after normalization with housekeeping gene *Ppia* ^10^. Standard error of mean (SEM) was measured from at least three independent experiments and represented as error bars.

### ChIP assays

To identify enhancers and SEs, ChIP assays were performed with enhancer-specific histone modifications as described previously^63^. Briefly, RVSMCs were cross-linked with 1% formaldehyde for 10 min at room temperature followed by the addition of 0.125 M glycine for 5 min to stop the reaction. Cross-linked cells were washed with ice-cold PBS and lysed in ChIP lysis buffer (4–8 × 10^6^ cells ml^−1^), supplemented with Protease inhibitors (Roche, Pleasanton, CA). The cell lysates were then sonicated using Bioruptor^R^ Pico (Diagenode, Denville, NJ) for six cycles (30 s on/30 s off) to shear the chromatin up to 200–600 bp. Sheared and pre-cleared chromatin (0.3 × 10^6^ cell equivalents) was used for each immunoprecipitation. About 10% of chromatin from each ChIP reaction was saved as input DNA. Immunoprecipitation was performed using 3–5 μg of antibodies specific to H3K27ac and H3K4me1 (Cat. Nos. ab4729 and ab8895, respectively; Abcam, Cambridge, UK), and BRD4 (Cat. No. A301-985A100; Bethyl, Montgomery, TX) overnight at 4 °C. Rabbit and mouse IgG were used for control ChIPs. Subsequently, 30 μl of magnetic protein G Dynabeads (Novex, Waltham, MA) were added to the ChIP reactions and incubated for 4 additional hours at 4 °C. Magnetic beads were washed well with wash buffers. Bound chromatin was eluted, DNA-protein cross-links were reversed, digested with Proteinase K and DNA was purified by phenol-chloroform extraction followed by ethanol precipitation. Resultant ChIP-DNA was dissolved in water. For ex vivo and in vivo ChIP assays, aortas were cross-linked with PBS containing 1% formaldehyde for 20 min at room temperature and quenched by addition of PBS containing glycine (0.125 M) for 5 min. The cross-linked tissues were subsequently washed with ice-cold PBS and lysed in 250 µl ChIP lysis buffer. The lysates were then sonicated using Bioruptor^R^ Pico for 12 cycles (30 s on/30 s off) to shear the chromatin up to 200–600 bp. Subsequent steps were same as described above for ChIP with RVSMCs.

### ChIP-qPCRs

ChIP-DNA was analyzed by qPCR with primers specific for indicated genomic regions. ChIP primers for specific enhancer and SE regions were designed using web-based PrimerQuest software (Integrated DNA Technologies (IDT), Coralville, IA). Primers were named after “Nearest nearby” putative target genes of the identified enhancers. The primer sequences are provided in Supplementary Table 1. For ChIP assays with tissues from in vivo mice experiments, qPCR primers were designed for corresponding orthologous enhancers on mouse genome identified using the liftOver tool^55^ (UCSC genome browser). Subsequently ChIP-enriched DNA fragments were analyzed by qPCR using SYBR Green reagent on the 7500 Fast Real-Time PCR system using technical triplicates. ChIP enrichment relative to input (Percent input) was calculated using the formula 2^−^(^Ct^_ChIP_^−Ct^_100% input_). Standard errors were measured from the three independent culture experiments and represented as error bars.

### ChIP-seq analysis of RVSMCs samples

Library preparation and high-throughput sequencing were conducted at City of Hope’s Integrative Genomics Core. Briefly, 10 ng of ChIP-DNA was end-repaired (Epicentre, Madison, WI), and a single adenosine was added using Klenow exo^-^ (New England Biolabs, Ipswich, MA), and ligated to adapter oligonucleotides provided by Illumina. The adapter ligated DNA was enriched by 12 cycles of PCR using 2× Phusion High-Fidelity PCR Master Mix with HF Buffer (New England Biolabs) using the universal primer P1.0 (5′-AATGATACGGCGACCACCGAGATCTACACTCTTTCC CTACAC GACGCTCTTCCGATCT-3′) and a barcoded index primer (5′-CAAGCAGAAGACGGCATACG AGAT NNNNNNGTGACTGGAGTTC-3′). PCR products were purified using 1.0× AmpureXP beads (Beckman Coulter, Beverly, USA). Quality of the purified libraries was verified using Bioanalyzer 2100 system with DNA High Sensitivity Chip (Agilent, Santa Clara, CA) and quantified with Qubit (Life Technologies, Grand Island, NY). The library templates were prepared for sequencing using cBot cluster generation system (Illumina, San Diego, CA) with HiSeq PE Cluster V3 Kit. Sequencing run was performed in paired end mode of PE100 on Illumina HiSeq 2500 platform with HiSeq SBS V3 Kits. Real-time analysis 2.2.38 software was used to process the image analysis and base calling.

### ChIP-seq data analysis

Raw sequences of each ChIP-seq sample were aligned to rat genome assembly rn4 using Novoalign version 2.07.05MT (http://www.novocraft.com). We used MACS2.0 (Model-based Analysis of ChIP-Seq) (https://github.com/taoliu/MACS) to identify genomic regions enriched for each of the ChIP samples including four H3K27ac samples (two control and two AngII treated), four H3K4me1samples (two control and two AngII treated), four BRD4 samples (two control and two AngII treated), and two previously published H3K4me3 samples^11^. We identified broad peaks^64^ with a *P* < 0.1 (based on Poisson distribution and other parameters used by the MACS2 software) which contain narrow peaks with *P < *0.05 for H3K27ac, BRD4, and H3K4me1 samples and narrow peaks for H3K4me3 using corresponding input sample as the control.

### Identification of differentially enriched enhancers

To profile enhancers genome-wide, regions with both H3K27ac and H3K4me1 enrichment (commonly enriched) were first identified. They are defined as regions containing peaks in at least two out of four samples for H3K27ac and similarly for H3K4me1^65^. Among these regions, the ones overlapping with promoters (±1000 bp from transcription start sites), exons of known genes, and H3K4me3 peaks (potential promoters) were subsequently excluded. For each of these enhancers, a number of paired reads were counted and scaled to 1 million total paired reads in each ChIP sample (represents as Count_ChIP_) and input sample (as Count_input_). The enrichment in each ChIP sample at each enhancer was then calculated as log_2_(Count_ChIP_ + 1)/(Count_input_ + 1). Quantile normalization was then applied to the resulting H3K27ac or H3K4me1 matrices containing log2 enrichment levels of all the enhancers across all the samples. For each enhancer, Limma package was finally used to compare H3K27ac or H3K4me1 enrichment levels between AngII-treated and control samples. The differentially enriched enhancers were identified as fold change ≥2 for H3K27ac relative to controls at a significance level of BH adjusted *P* < 0.05, moderated *t*-test.

### Annotation of enhancers to genes and lncRNAs

Differentially enriched enhancers were assigned to the nearby AngII-regulated RefSeq genes (see below) and previously published rat lncRNAs^11^ whose transcription start sites were within its ±250 kb flanking regions.

### RNA-seq analysis

For each sample, paired-end raw sequences were aligned to rat genome assembly rn4 using Tophat with RefSeq genes as reference genes. The reads located in each RefSeq gene (including all the transcripts) were counted by HTSeq-count^66^ to obtain gene level reads including all the exons of each transcript. EdgeR^67^ was then used to normalize the resulting gene-level counts using the trimmed mean of M-values method. Differentially expressed genes between AngII-treated and untreated samples (AngII-regulated genes) were identified as the ones whose coverage were at least 10 in either sample and fold change between the two samples no less than 1.5.

### TF binding site analysis

TF binding site analysis of differentially regulated H3K27ac-enriched enhancers (1541 upregulated and 1328 downregulated, fold change ≥2 and BH adjusted *P < *0.05) was performed using Peak-motifs software available online at Regulatory Sequence Analysis Tools (http://rsat.sb-roscoff.fr/) website. We used 5201 enhancers showing the lowest differences between AngII-treated and control samples (fold change ≤1.05) as background. Top 10 enriched de novo motifs with Bonferroni adjusted *P-*value (E) < 0.05 (binomial distribution based on background sequences) were further searched using JASPAR database (http://jaspar.genereg.net/) to identify known TF binding sites.

We also performed motif search analysis to identify TF binding sites in the enhancers which are either formed (de novo gained enhancers) or lost upon AngII treatment (de novo lost) from H3K27ac-enriched enhancers. Here, de novo gained enhancers were defined as the differentially enriched enhancers where AngII treated H3K27ac ChIP enrichment signals (ChIP vs. input) are more than 1.5 and Control H3K27ac ChIP enrichment signals are less than 1.1. Lost enhancers were regions where AngII-treated H3K27ac ChIP enrichment signals are less than 1.1 and Control H3K27ac ChIP enrichment signal are more than 1.5.

### Association between lncRNAs and enhancers

To assess the significance of the overlap of key lncRNAs with enhancers, 10,000 simulations of proximity measurements were performed on randomly placed lncRNAs and enhancers in the rat genome. Specifically, 466 lncRNAs and 42,076 enhancers were first placed randomly across the 2,718,881,021 bp genome and then the number of overlaps between lncRNAs and enhancers was assessed. From this analysis, the empirical *P-*value for the 211 enhancers overlapping with lncRNAs was <0.0001.

### Droplet digital PCR

ddPCR reactions (20 µl) were run using ddPCR Supermix for Probes (no dUTP) (Bio-Rad Inc., Hercules, CA), 50 ng of genomic DNA from either WT or *lncAng383* KO cells, forward and reverse primers (900 nM) (genotyping primers) and FAM and HEX-labeled probes (250 nM) (FAM-TGCTTGGTACTGTCTTCAGTTTCTTGTATCCTCC-BHQ and HEX-TCTAGCATCCATGTTGGGCAGCTCTC-BHQ). ddPCR reactions and 70 µl of droplet generation oil (Bio-Rad) were used for droplet generation using QX200 droplet generator (Bio-Rad). 45 µl of droplet/oil mixture was transferred to a 96-well plate and amplification was performed in a T100 thermal cycler (Bio-Rad) using the PCR conditions (95 °C for 10 min; 40 cycles of 94 °C 30 s, 59 °C 1 min, 72 °C 2 min, 98 °C 10 min at 2 °C/s ramp rate and 4 °C ∞). After amplification, the PCR plate was read using the QX200 droplet reader (Bio-Rad). Finally, data were analyzed using QuantaSoft Analysis Pro software (Bio-Rad) to quantify the WT and deleted droplets.

### Identification of SNPs overlapping with enhancers

Homologous regions of the human genome to the rat enhancers were first identified using liftOver tool^55^. GWAS SNPs (http://www.ebi.ac.uk/gwas/) overlapping these regions were filtered and SNPs associated with cardiovascular-related diseases were highlighted.

### Network analysis

IPA software (http://www.ingenuity.com/products/ipa) was used to perform network analysis on the set of genes that were enhancer-associated and differentially expressed upon AngII treatment to identify enriched canonical pathways, molecular functions, and biological processes (BH adjusted *P* < 0.05).

### GREAT analysis

Functional annotation of H3K27ac enhancers (upregulated and downregulated) was performed using GREAT (http://bejerano.stanford.edu/great/public/html/input.php) tool. Since, GREAT tool does not support rat genome, orthologous mouse enhancers were identified using the liftOver tool^55^ from UCSC genome browser and analyzed with the Basal plus extension association rule using whole mouse genome as background^36^.

### Gene set enrichment analysis

The log2 fold change values of differentially expressed genes associated with AngII-upregulated enhancers was used for pre-ranked GSEA^35^ using the biological process and TF gene sets. Significantly enriched gene sets (empirical *P* < 0.05 based on gene sets permutation (*n* = 1000)) were used to generate a bubble plot with differentially regulated genes (absolute log_2_ fold change ≥0.5). The size of the bubble represents the number of genes with altered expression within each gene set and the axes represent the average log2 normalized gene expression.

### Identification of SEs

SEs were identified using ROSE^22,41^ algorithm, using enhancers defined as regions overlapping H3K27ac and H3K4me1 for ctrl and AngII data sets. For BRD4 data sets, enhancers were defined as BRD4 peaks identified using MACS2 (see ChIP-seq data analysis section).

### AngII gained and lost SEs

AngII gained and lost SEs were identified as described earlier^23^ using H3K27ac signals. In brief, background subtracted ChIP-seq signal (reads per million) of H3K27ac was calculated at all the enhancers considered SE in at least one condition. Gained SEs were defined as those with fold change in signal ≥2 after AngII treatment and lost SEs were defined as those with fold change ≤0.5 after AngII treatment. Similarly, background subtracted ChIP-seq signal of BRD4 was also calculated for the AngII gained and lost SE regions.

Heatmaps for the H3K27ac and BRD4 occupancy at the differential SEs and at promoters of proximal genes were made using NGS plot^68^.

### Motif analysis from AngII gained and lost SEs

Motif analysis was performed using HOMER^69^ with SEs gained or lost upon AngII treatment as input. Both de novo and known motifs were considered.

### Expression of genes proximal to AngII gained/lost SEs

Each SE or TE identified was mapped to the closest gene ±50 kb from the center of the enhancer using the ROSE geneMapper script from ROSE package. Genes that had less than 100 A.U. (arbitrary unit) normalized expression values were filtered out. Genes proximal to both a SE and a TE were assigned to SEs. Expressed genes (>100 A.U.) that did not map to any enhancer (±50 kb) were labeled “no enhancer genes”.

### Enhancer reporter plasmids and enhancer activity assays

To analyze enhancer activity, pGL4.10[luc2] vector (Promega) was modified to place firefly luciferase reporter gene under the control of rat *Ccl2* promoter. Briefly, a 615 bp minimal Rat *Ccl2* promoter fragment containing 550 bp sequence upstream from TSS and multiple cloning sites (SfiI, KpnI, BglII, NheI, XhoI, EcoRV, and NotI) at the 5′-end and a HindIII site at 3′-end was synthesized as a gBLOCK (IDT) and cloned into SfiI-HindIII sites of the pGL4.10[luc2]. Then, rat enhancer fragments were cloned upstream of the *Ccl2*-promoter in the resultant pGL4.10[luc2]-Ccl2 vector using SfiI and NheI sites. For *Spry2* and *Esm1A* enhancer cloning, the respective enhancer sequences were synthesized with NotI restriction site at 3′-end as gBLOCK, digested with NotI, end-filled with Klenow and subsequently cloned into the EcoRV site of pGL4.10[luc2]-Ccl2 vector. Clones were confirmed by restriction digestion and DNA sequencing. RVSMCs were co-transfected with these enhancer reporter constructs and a pRL-TK plasmid expressing Renilla luciferase (internal control) using Nucleofection method as described before^10^. Cells were incubated for 24 h, followed by serum depletion for further 24 h and then treated ± AngII for 6 h. Luciferase activity in cell lysates was measured using the Dual-Luciferase Reporter Assay System (Promega, Madison, WI). The primer sequences used for cloning enhancer regions are listed in Supplementary Table 1.

### DsiRNA transfection

RVSMCs were transfected with DsiRNAs targeting indicated genes and DsiNTC control oligos (IDT) using Nucleofection method as described before^10^. Cells were incubated for 24 h, serum depleted for further 24 h and then treated ± AngII for 3 h followed by RNA extraction for gene expression analysis. DsiRNA sequences are listed in Supplementary Table 1.

### CRISPR-Cas9-mediated deletion of enhancer and SEs

Target-specific CRISPR single guide RNAs (sgRNAs) for enhancer and SE deletion were designed using an online tool (http://crispr.mit.edu/) taking into consideration their uniqueness and limited off-target effects. Oligonucleotides corresponding to the sgRNA sequences as well as their complementary sequences were procured from IDT. Subsequently they were annealed and cloned into the BbsI site of the pSpCas9(BB)-2A-Puro (PX459) V2.0 (from the Feng Zhang Laboratory, Addgene plasmid #62988) following published protocols^70^. The pX459 plasmid expresses a human codon-optimized SpCas9 in addition to the guide RNA and puromycin resistance which is used for selection of transfected cells. The sgRNA sequences are enlisted in Supplementary Table 1. The CRISPR-Cas9 constructs were confirmed by BbsI restriction digestion and sequencing.

RVSMCs were transfected with the corresponding pair of CRISPR-Cas9 constructs for each deletion. Briefly, 1 × 10^6^ cells were transfected with 1 µg of each CRISPR plasmid using Nucleofection method. 24 h post transfection, culture media was supplemented with puromycin (1 µg ml^−1^) for 4 days in order to select for transfected cells.

Genomic DNA was isolated from the heterogeneous population of puromycin selected RVSMCs using QuickExtract^TM^ DNA Extraction Solution 1.0 (Epicentre). To screen for deletion of enhancer and SEs, primers were designed to regions that flank the deletion region, outside of the CRISPR sgRNAs. The PCR primer sequences are listed in Supplementary Table 1. PCR was performed using 20 ng of genomic DNA using deletion-spanning primers and Phusion High-Fidelity PCR Master mix (NEB) and reactions were run in agarose gels. Specificity of PCR reactions was confirmed when a unique band of expected size was observed in the WT RVSMCs DNA. Whereas deletion of enhancers and SEs was confirmed when multiple smaller deletion bands were observed in addition to the specific WT band in knockout RVSMCs.

The expression levels of the enhancer or SE-associated neighboring genes were assessed by treating WT or enhancer/SE deleted RVSMCs ± AngII and performing RT-qPCR, as described earlier.

### Statistical analysis

All in vitro experiments with RVSMCs were performed at least three times unless indicated otherwise. Based on our previous experience and power analysis, we estimated that a sample size of seven mice per group can provide 93% power to detect an effect size of 2 at 0.05 significance level using two-tailed *t*-test. Therefore, we used at least seven mice per group for the in vivo experiments. Data are represented as mean and standard error (mean + SEM). Statistical analyses were performed using GraphPad Prism 7.02 software (GraphPad Prism Software Inc., San Diego, CA) unless stated otherwise. Normal distribution of each sample group was confirmed by Shapiro–Wilk normality test before comparing groups. For statistical comparison of two groups, we used unpaired two-tailed Student’s *t*-test. Welch’s *t*-test was used if variances between two groups were significantly different (*F*-test). For the comparison of three or more groups, as variances were similar among groups being compared, we used one-way ANOVA followed by Tukey’s or Dunnett’s post hoc tests as indicated. *p*-values < 0.05 were considered statistically significant for all tests used.

### Data availability

The authors declare that all the data supporting the findings in this study are available in this study and its Supplementary Information, or are available from the corresponding author through reasonable request. The sequencing data sets have been deposited in the NCBI Gene Expression Omnibus (GEO) database under accession number GSE95067.

## Electronic supplementary material

Supplementary Information
