# Supplementary material for: Regulation of angiotensin II actions by enhancers and super-enhancers in vascular smooth muscle cells
Source: Nat Commun. 2017 Nov 13;8:1467. doi: 10.1038/s41467-017-01629-7 (PMC5684340; doi:10.1038/s41467-017-01629-7)
Supplement: Supplementary file 1 — Supplementary Information [file 41467_2017_1629_MOESM1_ESM.pdf]

Supplementary Figures and Tables

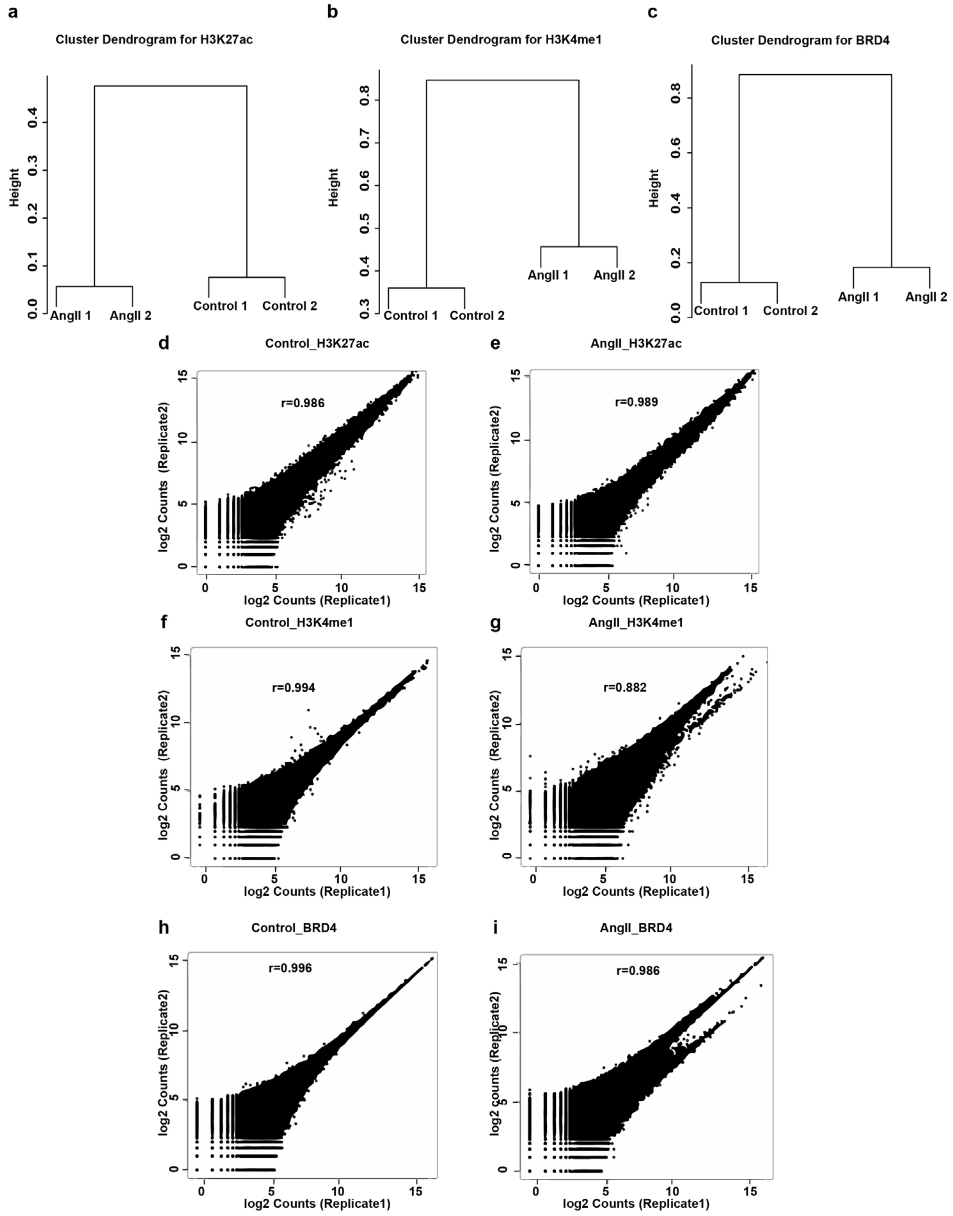

Supplementary Figure 1. Correlation between ChIP-seq replicates. **(a)** Cluster analysis of H3K27ac. **(b)** H3K4me1. **(c)** BRD4 ChIP-seq. Rat genome was divided into 566848 non-overlapping 5-kb windows. For each sample including both ChIP and input samples, coverage was calculated on each window and scaled to total number of reads across all the samples. Log2(ChIP/input) was then calculated for each window. The windows with enrichment levels (log2(ChIP/Input) < 2 in more than 2 samples were excluded. The dissimilarities, defined as 1-cor, from the resulting data were finally calculated across the 4 samples (2 AngII and 2 controls) which were subjected to cluster analyses and shown as dendrograms. **(d-i)** Scatterplots of read counts (log2 counts) on identified peak regions for each replicate of H3K27ac, H3K4me1 and BRD4 ChIP-seq data for Control and AngII samples as indicated. Data shows very good correlation between the replicates (Pearson correlation coefficient (r)=0.0882-0.996).

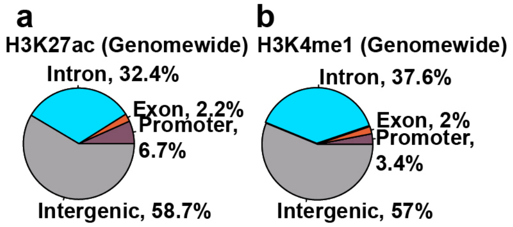

Supplementary Figure 2. Genomic distribution of enhancer marks in VSMC. Venn diagrams depicting **(a**) Distribution of H3K27ac and **(b)** H3K4me1 in the indicated genomic regions.

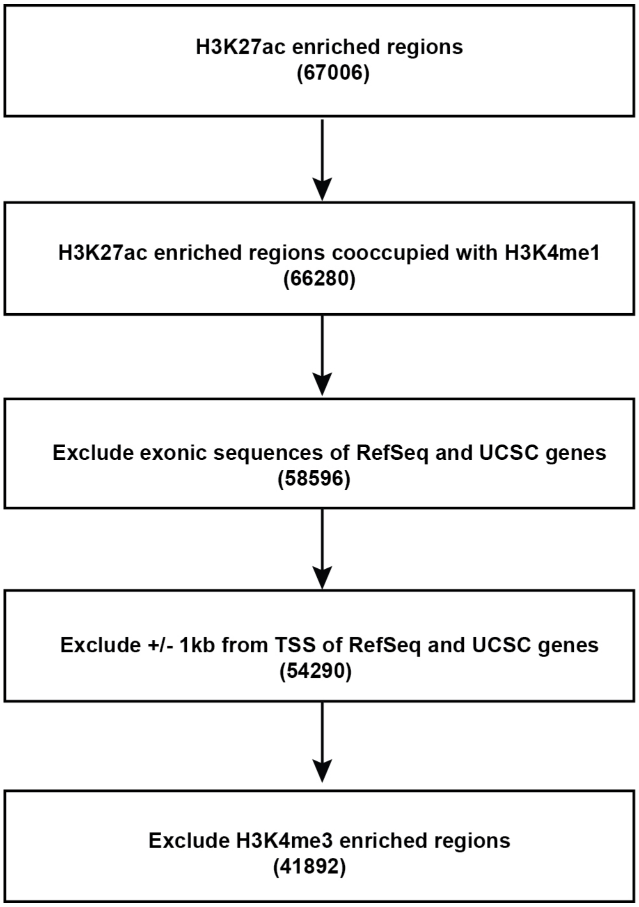

Supplementary Figure 3. Strategy for enhancer identification. Briefly, H3K27ac enriched regions were identified and filtered based on co-occupancy of H3K4me1. H3K27ac and H3K4me1 co-occupied regions were used to filter out exonic and promoter sequences of RefSeq and UCSC genes followed by removal of H3K4me3 enriched regions.

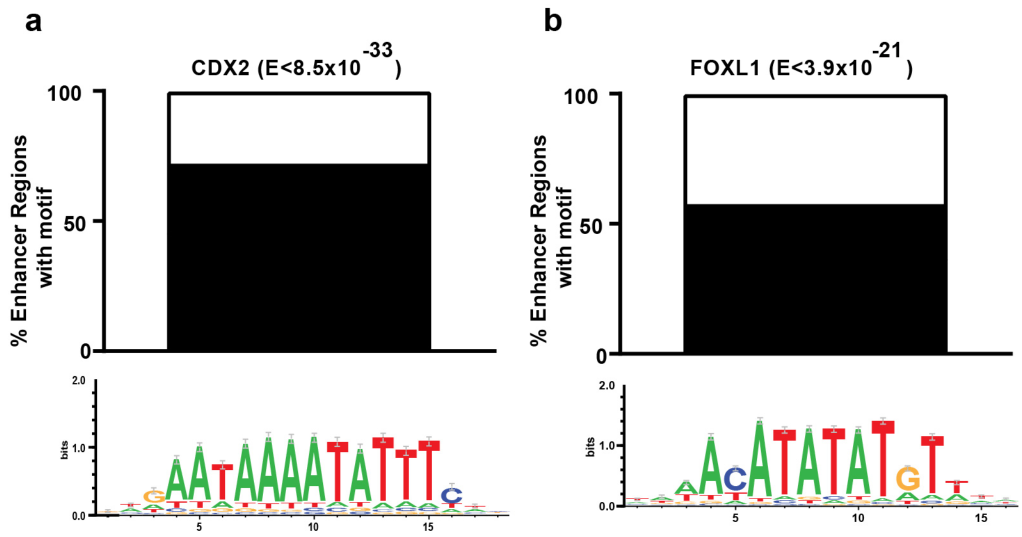

Supplementary Figure 4. Motif discovery from AngII-downregulated enhancers. **(a-b)** Enrichment of CDX2 and FOXL1 motifs in AngII-downregulated enhancers.

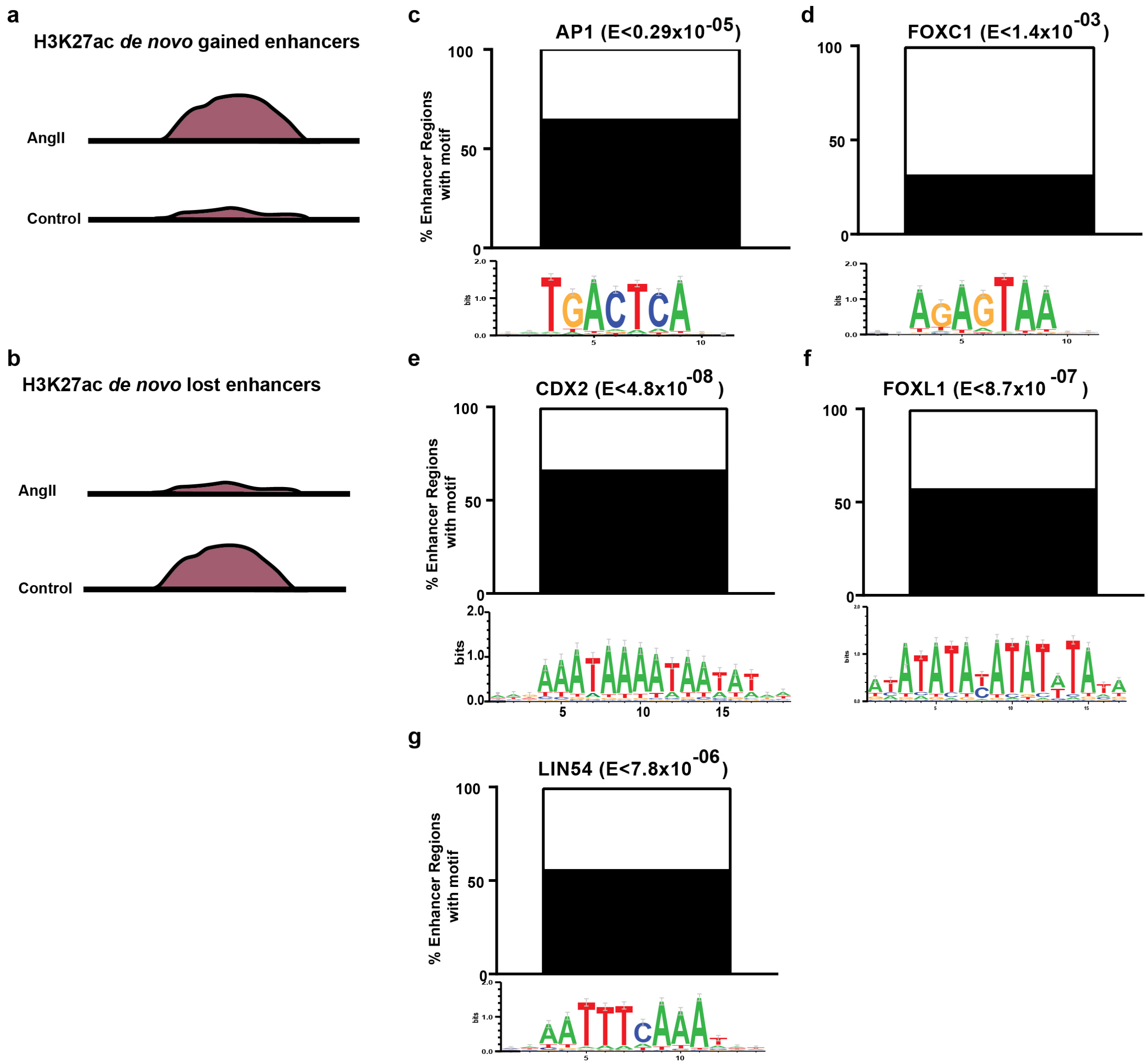

Supplementary Figure 5. Enrichment of TF motifs on *de novo* gained and lost enhancers. **(a)** Schematic represents formation of *de novo* H3K27ac gained enhancers by AngII. **(b)** Schematic represents loss of H3K27ac enhancers by AngII. **(c-d)** Representative motifs of AP1 and FOXC1 are statistically enriched in H3K27ac-gained enhancers (where AngII treated H3K27ac ChIP enrichment signals are more than log2(1.5) and Control H3K27ac ChIP enrichment signals are less than log2(1.1)) and shown as in % of enhancers with individual motif. **(e-g)** Representative motifs of CDX2, FOXL1 and LIN54 enriched in H3K27ac lost enhancers (where AngII treated H3K27ac ChIP enrichment signals are less than log2(1.1) and Control H3K27ac ChIP enrichment signals are more than log2(1.5)) and shown as % of enhancers with individual motif. E is Bonferroni adjusted *P* value.

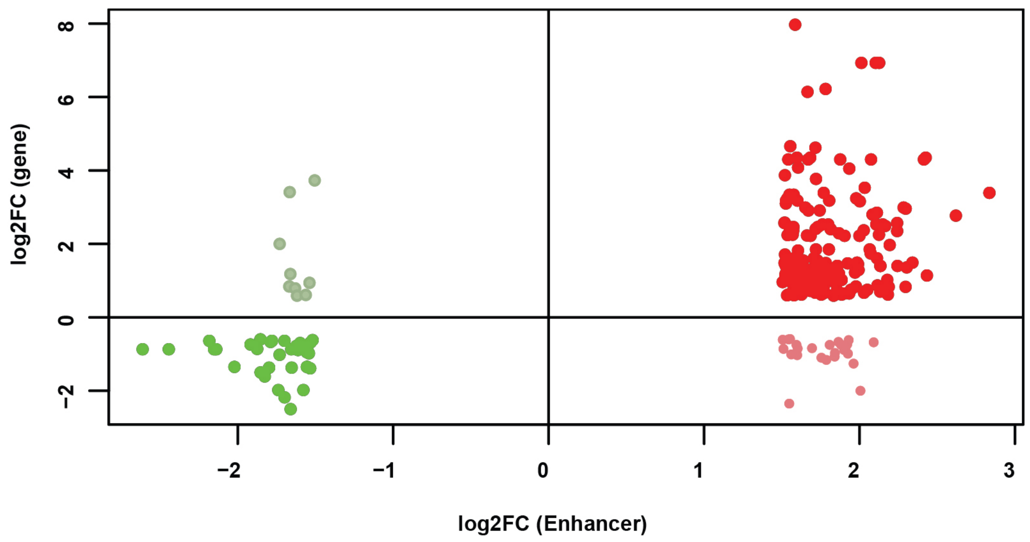

Supplementary Figure 6. Correlation between differentially enriched enhancers and differentially regulated genes. Scatter plot showing the log2 fold change in expression of genes associated with differentially enriched H3K27ac enhancers (-1.5 > log2FC > 1.5). Genes associated with upregulated enhancers are colored in shades of red and those associated with downregulated enhancers are colored in shades of green.

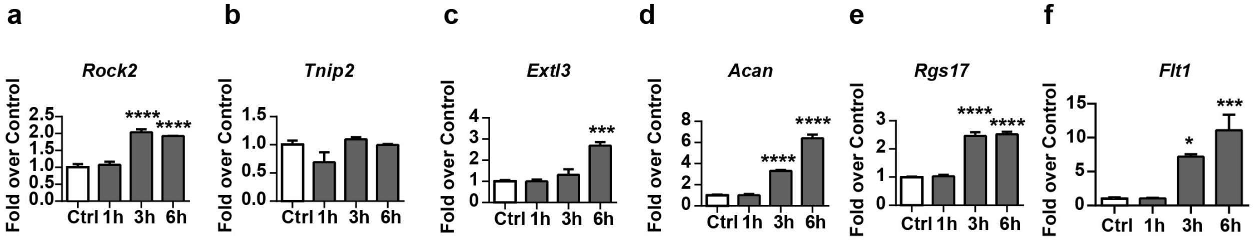

Supplementary Figure 7. Enhancer-associated neighboring gene expression. (a-f) Expression of the indicated genes were validated in RVSMCs treated ±AngII for 1-6 hrs *in vitro.* Mean+SEM; *P<0.05; ***P<0.001; ****P<0.0001; vs. Ctrl; n=3, using one-way ANOVA, Dunnett's multiple comparisons test.

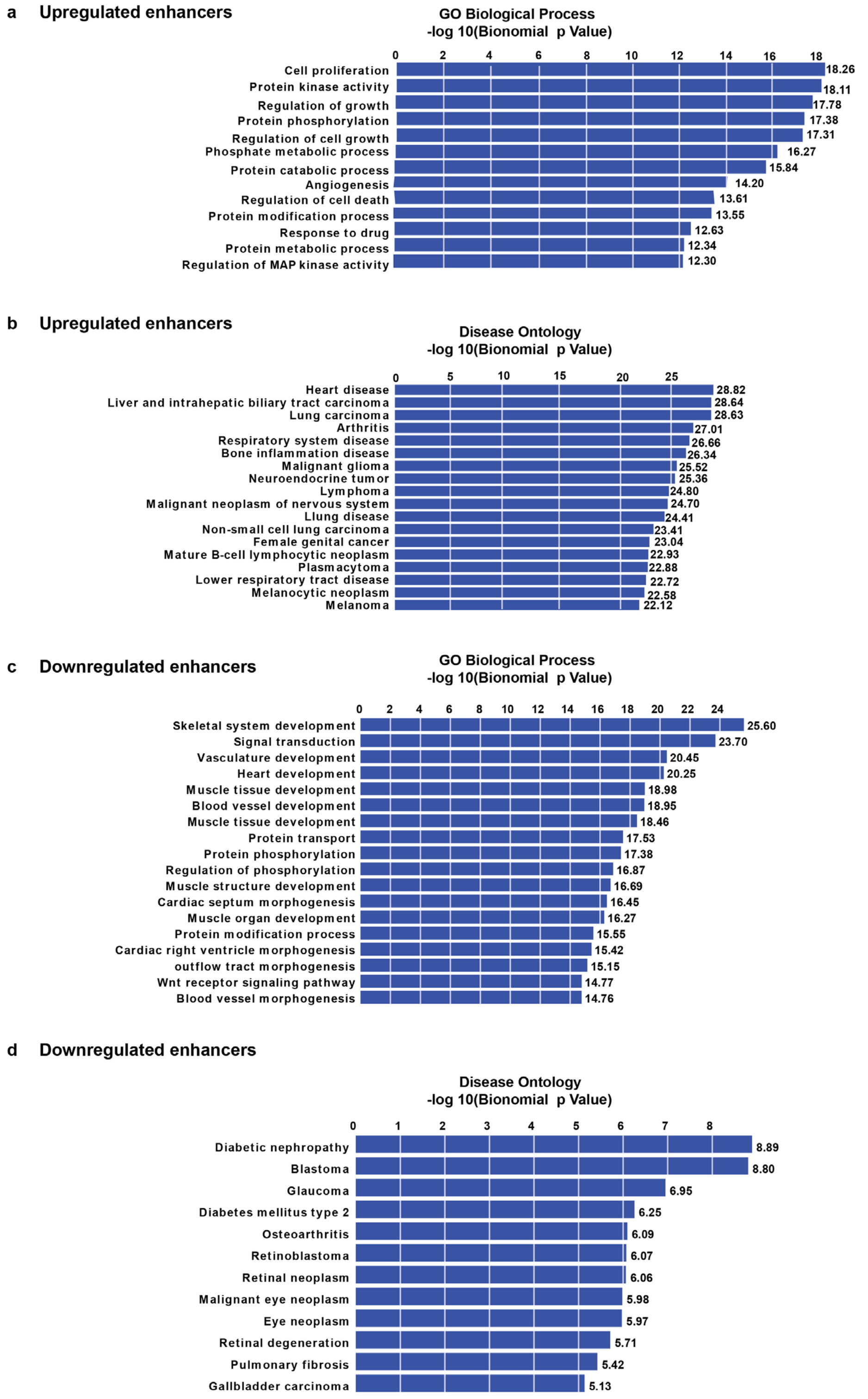

Supplementary Figure 8. Functional annotation of differentially regulated enhancers. (a-d) Identification of GO biological processes and Disease Ontology using GREAT tool. Upregulated enhancers are shown in panels **(a)** and **(b)**, whereas downregulated enhancers are shown in panels **(c)** and **(d)**. Values represent -log10 (Binomial *P* value).

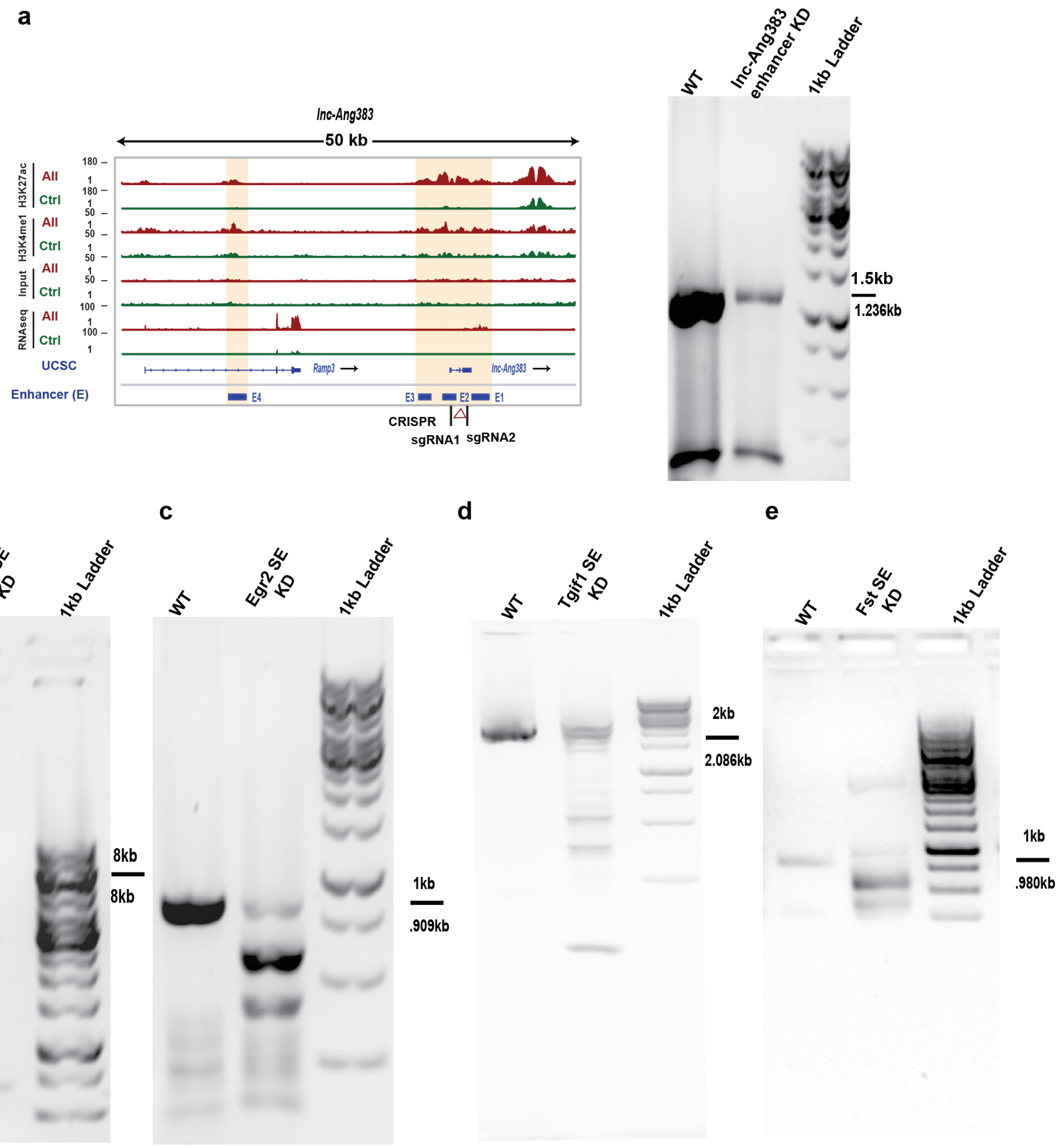

Supplementary Figure 9. CRISPR-Cas9 mediated deletion of candidate enhancers/SEs. **(a)** Genome browser tracks showing the relative position of the indicated enhancers (highlighted in orange) with respect to nearby indicated gene and the position of the sgRNAs (denoted by black lines) designed to delete the enhancer overlapping with lncRNA, *lnc-Ang383* using CRISPR-Cas9 editing in RVSMCs, and genotyping PCR result of lnc-Ang383 enhancer deletion. **(b-e)** Genotyping PCR results to confirm deletion of SEs for *Fgf2* **(b)**, *Egr2* **(c),** *Tgif1* **(d)**, and *Fst* **(e)**. The expected band size for each PCR is indicated on the right side of each figure.

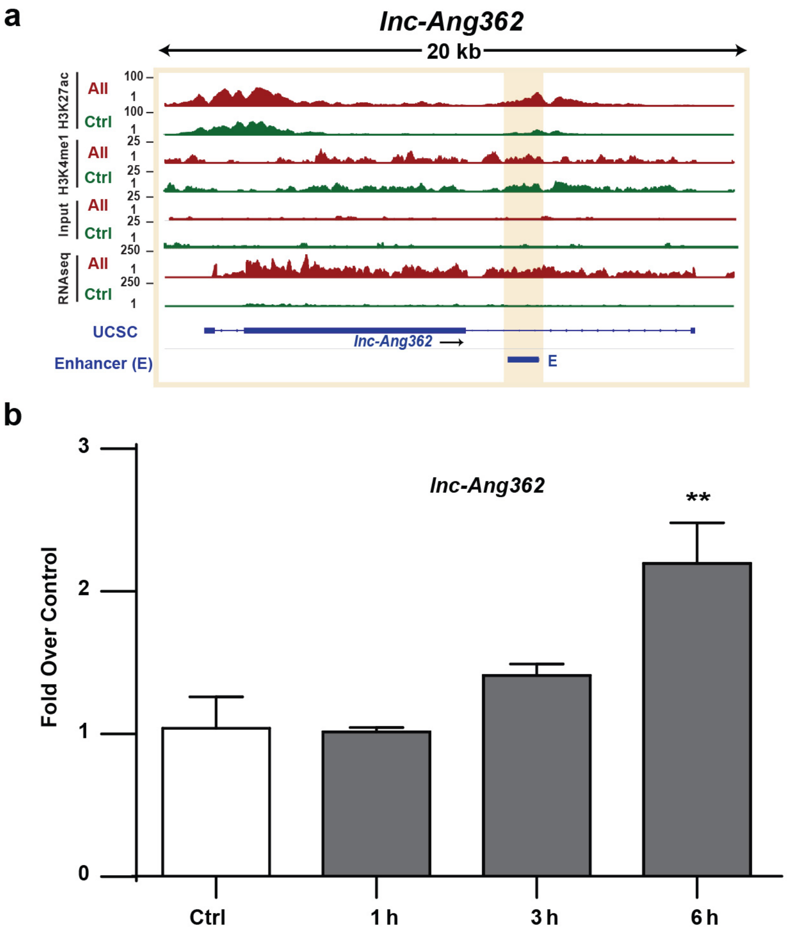

Supplementary Figure 10. Genomic organization of *lnc*-Ang362 overlapping enhancer and its regulation by AngII. (a) Tracks show profiles of H3K27ac and H3K4me1 signal on RVSMC specific enhancer associated with *lnc-Ang362*. Each data track shown is on the same scale for both Control (Ctrl) and AngII. **(b)** Bars represent fold change in expression of *lnc-Ang362* upon treatment of RVSMCs with AngII for the indicated times. Mean+SEM; $^{**}P<0.01$; vs. Ctrl; $n=3$, using one-way ANOVA, Dunnett's multiple comparisons test.

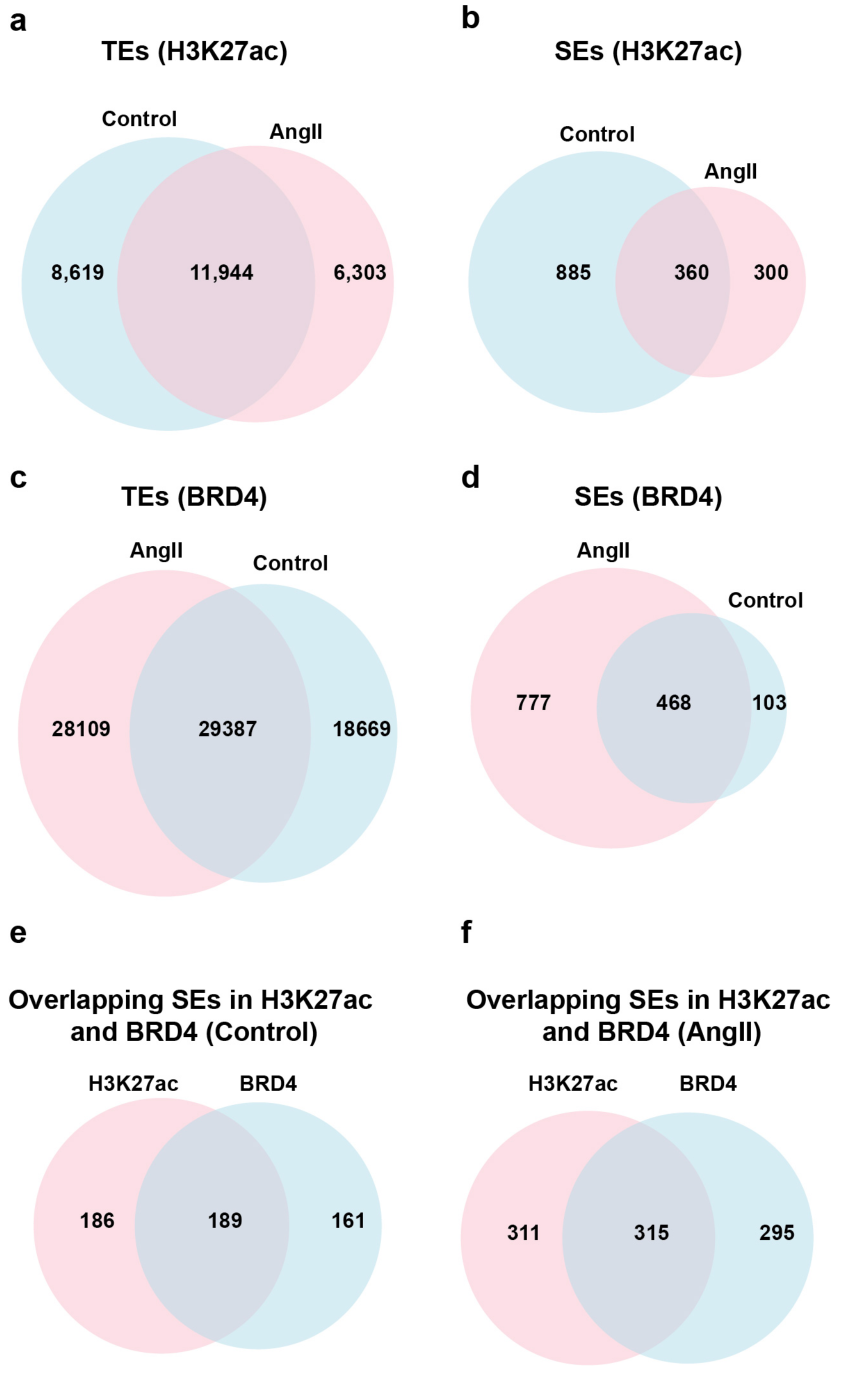

Supplementary Figure 11. Comparison of H3K27ac and BRD4 specific TEs and SEs. (a) Venn diagram showing the overlap between H3K27ac TEs and **(b)** SEs identified in Control and AngII conditions. **(c)** Overlap between BRD4 TEs and **(d)** SEs identified in Control and AngII conditions. **(e)** Overlap between H3K27ac and BRD4 SEs identified in Control (untreated) and **(f)** AngII treated RVSMCs.

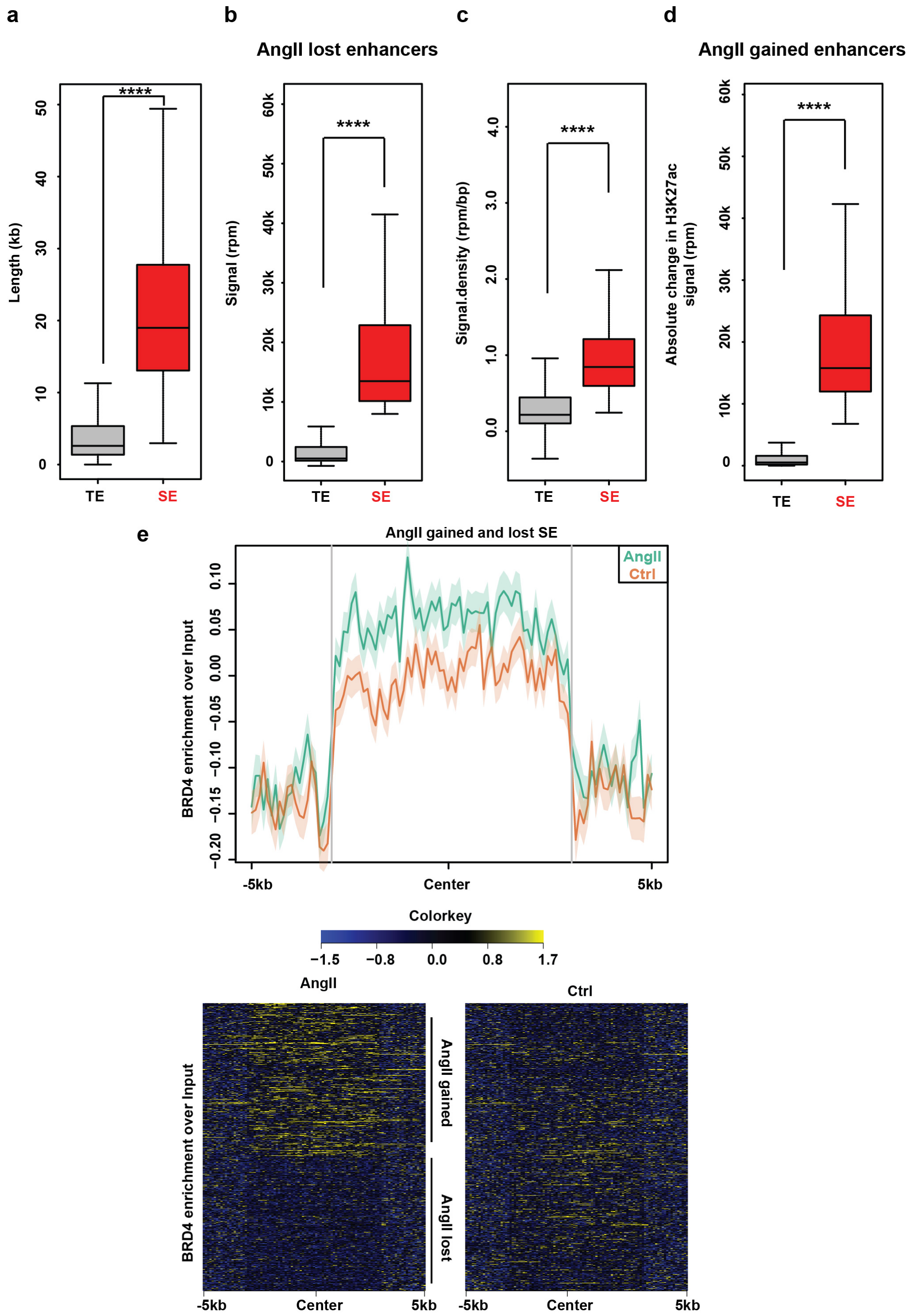

Supplementary Figure 12. Characterization of AngII gained and lost SEs. (a) Boxplots show median enhancer length (kb), **(b)** signal (rpm), and **(c)** density (rpm/bp) in AngII-lost TEs and SEs. **(d)** Boxplot shows the absolute change in H3K27ac signal in response to AngII treatment measured at AngII gained TEs and SEs. For **(a-d)**, ****$p<0.0001$ using unpaired two-tailed t test. **(e)** Average profile and heatmaps of BRD4 enrichment over SEs gained and lost upon AngII treatment.

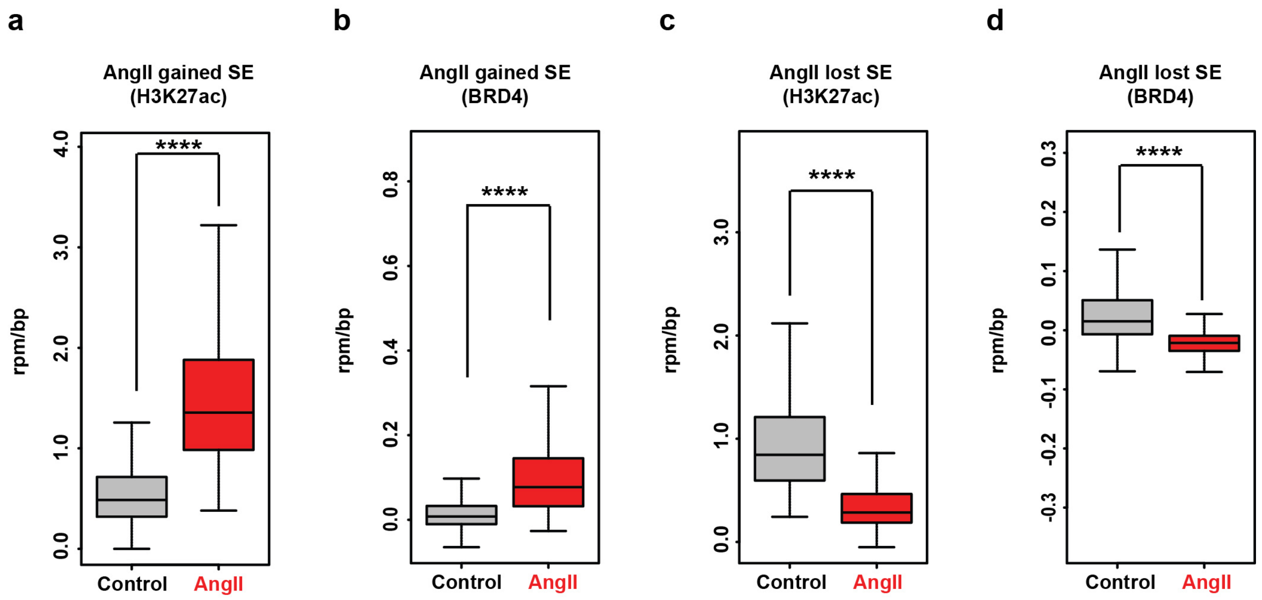

Supplementary Figure 13. **AngII-induces co-ordinate changes in H3K27ac and BRD4 occupancy over SEs. (a)** Boxplot of H3K27ac and **(b)** BRD4 signal density (rpm/bp) at AngII gained SEs. **(c)** Boxplot of H3K27ac and D. BRD4 signal density (rpm/bp) at AngII lost SEs. ****P<0.0001, using unpaired-two tailed t test.

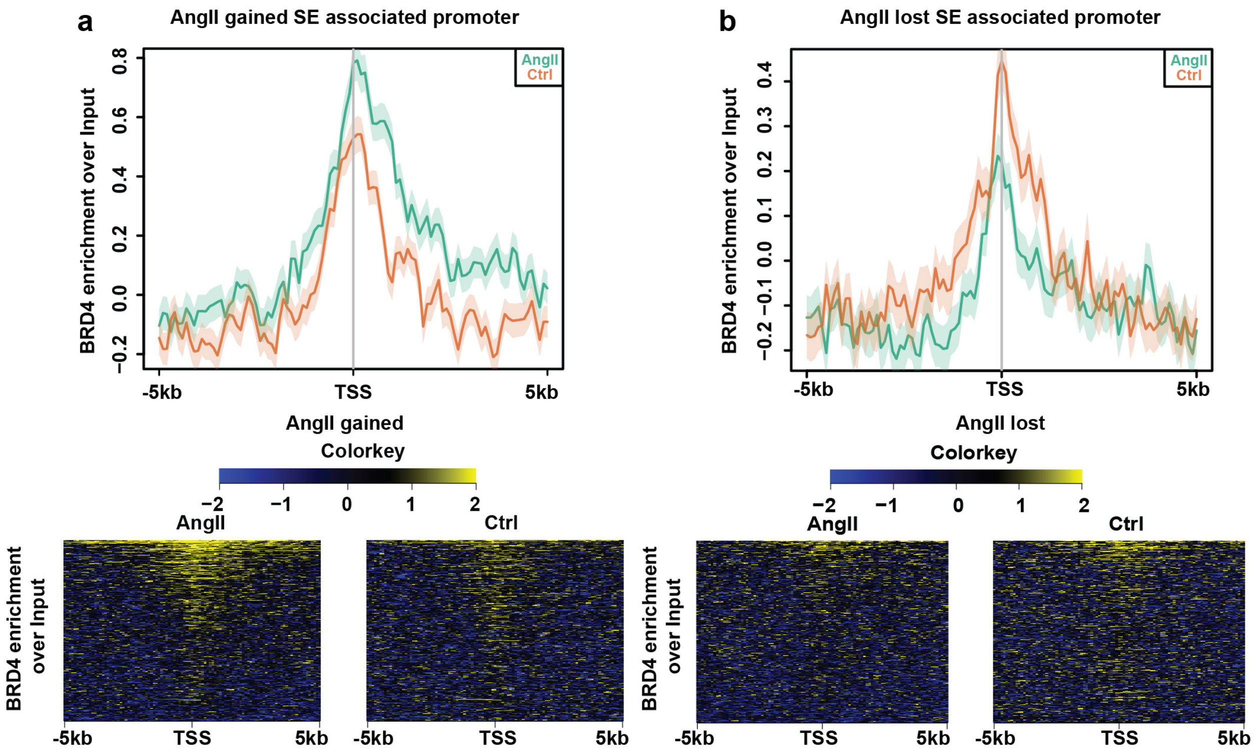

Supplementary Figure 14. BRD4 enrichment mirrors H3K27ac enrichment on SE regions. **(a)** Average profile and heatmaps of BRD4 enrichment over promoters of AngII gained and **(b)** AngII lost SE-associated genes.

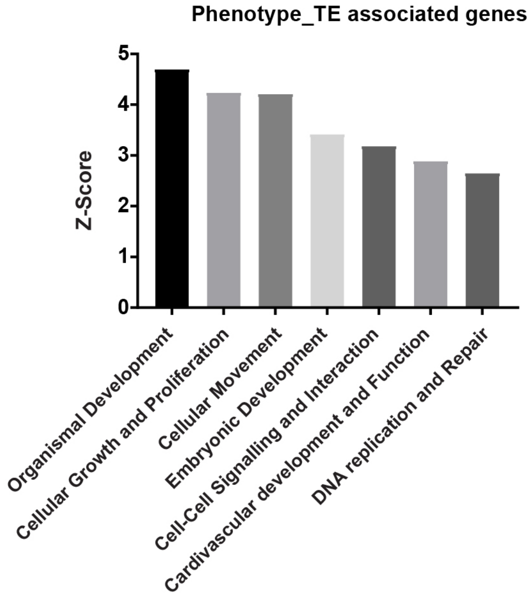

Supplementary Figure 15. Phenotypes associated with TEs. IPA Pathway analysis of genes associated with TEs.

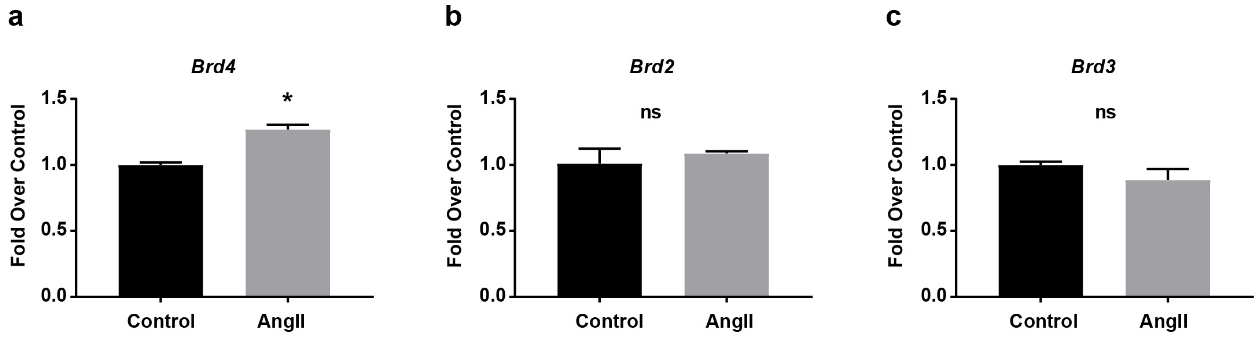

Supplementary Figure 16. Expression of BET family proteins in VSMCs. (a-c) *Brd4*, *Brd2* and *Brd3* expression were quantified by RT-qPCRs in RVSMCs treated ±AngII for 3 hrs *in vitro.* Mean+SEM; **P*<0.05; vs. Control; *n*=3, using unpaired-two tailed t test.

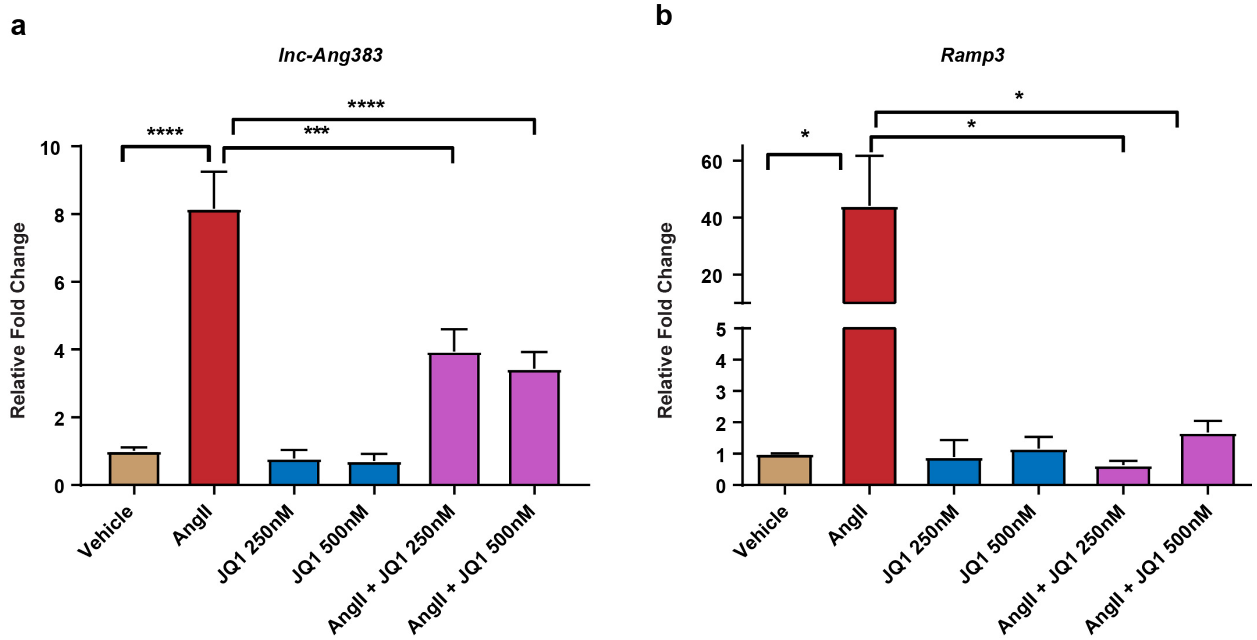

Supplementary Figure 17. JQ1 attenuates AngII-induced expression of *lncAng383* and *Ramp3*. (a-b) Bars represent the expression of SE-associated genes measured by RT-qPCR in RVSMCs pre-treated with vehicle (DMSO) or the BRD4 inhibitor JQ1 (250 and 500 nM) and then treated *in vitro* with AngII as indicated. Gene expression was normalized with *Ppia* and represented as relative fold change with respect to Vehicle. Mean+SEM; *P<0.05; ***P<0.001; ****P<0.0001; n=3, using one-way ANOVA, Tukey's multiple comparisons test.

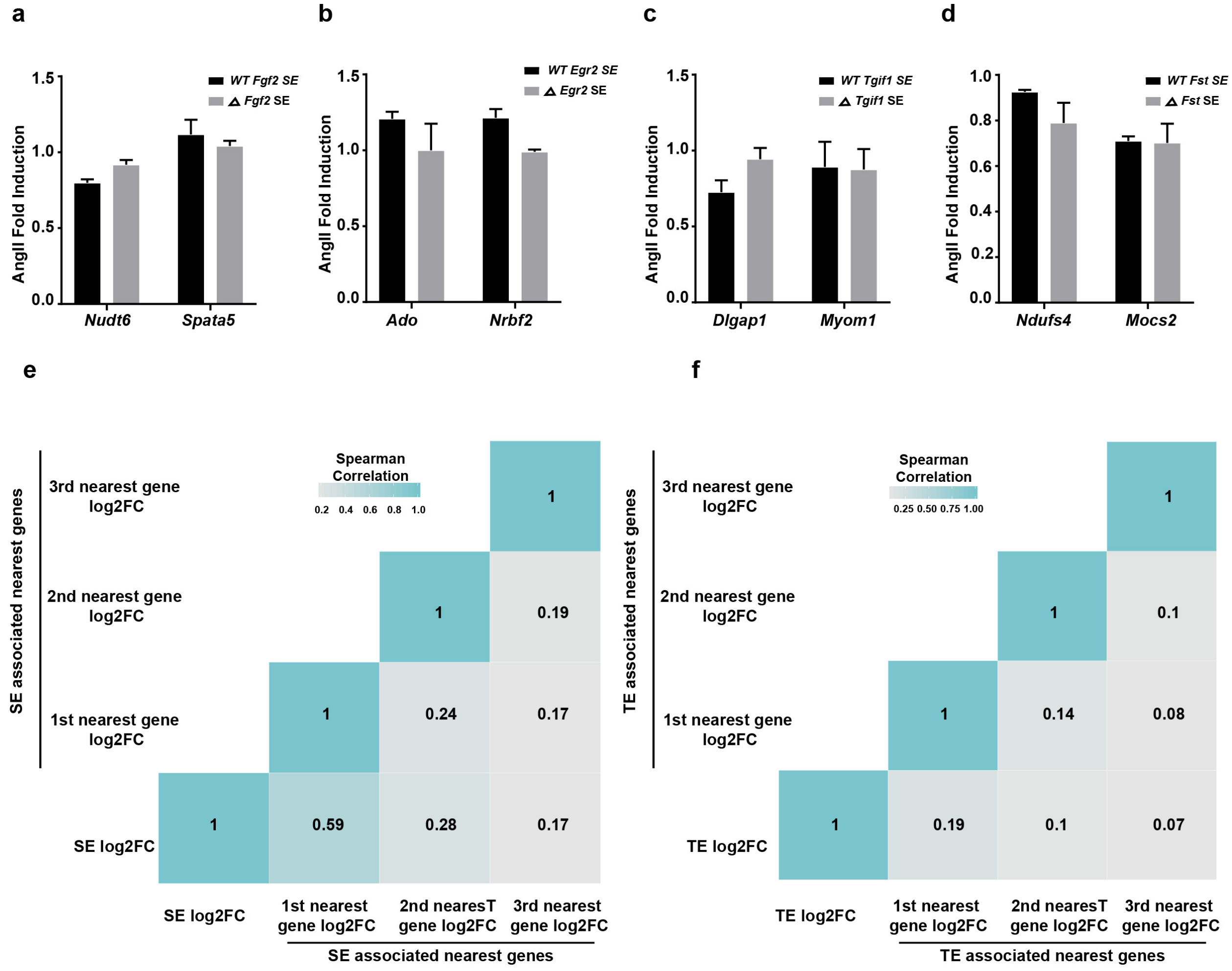

Supplementary Figure 18. SEs regulate the first nearest AngII-regulated genes. (a-d) Bar graphs show the AngII-induced expression of indicated genes in WT and SE deleted (ΔSE) RVSMCs. Gene expression was normalized to *Ppia* and expressed as AngII response vs. respective Controls. Mean +SEM, *n*=3. Data is not significant, using unpaired two-tailed t tests. **(e)** Correlation heatmaps representing Spearman correlation co-efficients between log2 fold change in H3K27ac occupancy at SEs and **(f)** TEs and the expression changes of 1st, 2nd and 3rd nearest gene mapped within ± 500 kb from the enhancer centre. For **(e-f)**, all differentially regulated enhancers (SEs/TEs) that had 1st, 2nd and 3rd nearest genes mapped within ± 500 kb from the enhancer centre were considered regardless of whether the nearest genes were AngII-regulated or not.

qRT-PCR expression

Refers to Figure 1 G-J

Gene	Forward Primer (5'-3')	Reverse Primer (5'-3')
Jun	agggttaagacaccgcttg	ggagaacctctgtcgctg
fos	ctccgtttctcttcctcttcag	tccttacggactccccac
Ets1	ctctcaactggcaggaagatg	gctcgtcaggtaggatttcag
Ets2	acggatgggaattcaagctc	tgcccgatgtcttgtgaatg
Ppia	ccgctgtctcttttcgcc	gctgtctttggaactttgtctg

Refers to Figure 2

Gene	Forward Primer (5'-3')	Reverse Primer (5'-3')
Esm1	cttccccttcttccagtatgc	gttccggtctccaatctcttc
Spry2	atcagatcttggcggtgtg	tgtgtaccttgctgttggag
Agtr1a	ggcagtcatcttggattcttttg	acacaaccctcccagaaag
*mEsm1**	ggaagatgtctggactttccc	cgttcccttctccaatctcttc
*mSpry2**	atcaggtcttggcagtgtg	agaggattcaagggagaggg
*mAgtr1a**	gctggcattttgtctggataac	gcttttctgggttgagttgg
*mPpia**	atggtcaaccccaccgtg	ttcttgctgtctttggaactttgtc

Refers to Figure 3

Gene	Forward Primer (5'-3')	Reverse Primer (5'-3')
Fgf7	tgaatactatcttgccatgaacaaag	acgaacatttcccctccg
Slc29a2	tggtgaaggtggatttgagc	tgaggaagagggtgctgtag
Serpine1	cacaagcactacaaaaggtcaag	gccgaaccacaaagagaaag
CD44	cttccagctctccaggtaatg	ctccagtcatagtacaacccttc
Egr3	attacactcagatggctacagc	ctggaaagagcccgaataagag

Vcl	aggagaaacagaaaagagggc	ctgtaacttcctaacctttttcacatg
Clu	tcttggcacttctcacactg	agctttacagttcccggatg
Foxp1	gttttgtgcgagtagagaacg	cattgagaggtgtgcagtagg

Refers to Supplementary Figure 7

Gene	Forward Primer (5'-3')	Reverse Primer (5'-3')
Rock2	cacaagaccacaaaagcacg	gagtaggttctccactttctgc
Tnip	ctgatgagacccaccaactg	ggtatccctgcttgtatgctc
Extl3	accccacaatgcgatcac	cttgccctggaatgagatagag
Acan	aaccttcgctccaatgactc	ctcggtcaaagtccagtgtg
Rgs17	Cccacacaccaccaaaatg	gagaccaggacaagacttcatc
Flt1	cctcagcctaccatcaagtg	gccctcgattctgtttcctatg

ChIP Primers

Refers to Figure 3

Enhancer proximal to	Forward Primer (5'-3')	Reverse Primer (5'-3')
Neg Ctrl	agcttaaagtgtctccagtcaa	atatcaggctcctttgtgaagt
Spry2	actttctgacagagctggttatt	tgaggtgtctcctggaatct
FGF7	tccttcttccattgggtgattt	ggtacagagacaggcagataga
Slc29a2	ggtcctcgacaagaacagaatag	taggaggaggaaggctcaaa
Serpine1	cttactggaagcgaggtgttag	gagacagcacttagaggaatgg
Sgk	ccagactacctgcctattaatctc	cctgagctgagtgtatgtaagg
CD44	gcagggatgacacatcagaa	gttggtcgtggtgtctcttta
Ezr	cctagtcaggttctgtctgtct	tgttctgtgttcccttgtcttac
Esm1A	acatacaggcactcacagaac	gcatggcaaactcagaatcac
Esm1B	agcaccaggtaaccaactattc	ctcttggagagtttcccttctaac
Esm1C	gaaccagaacatcagggaagtc	cagcgtctggcaagtagtaatc

Egr3	ctgtgtatctggcatacgatctg	ctccgtgtgtgagtatgtgtatag
Cebpg	ctgaagtggaaggactgaactg	gtggtgggcagaatgagaaa
Jun	tggatggatggcacagttac	agagagagagagagagagagaga
Foxp en1	tgtctcttcagggcaacataac	aagctgttgggttccttctatc
Foxp en2	ctccttaccttccctttctgttt	ggtcccttggtcactgttatg
Ets2	cagtgagttctgcctacttctg	caaggtggagacagggttatg
Agtr1aUA	cgactggttgccttgtatct	ctagggacccagactgaataaac
Agtr1aUB	caggtgccactggatacaaa	accctcacctagatcagtaagt
Agtr1aDA	ctgacagactactctgcaatcc	ctgccttgggatctctcataaa
Agtr1aDB	caagtgcccatacctactactg	ggctaatccctcgtcttctatg
Ago2	tggcaggtccttattgctattc	gggtctcctagagatggacttac
Vcl	aacaggcaggagcaaaca	gtacgtgtaggtttccgtgtag
Clu	cttagctccattccctgacaaa	tctccttcccagccctaaa
mEsm1A*	ttgccaccttgaggactattg	gcctgccttcctctgtaaat
mEsm1B*	cccgtgggtgaatgagatatt	agcgtagggtgagacaaatg

Luciferase Constructs

Refers to Figure 4

Construct	gBLOCK sequence (5'-3')
Ccl2-Prom	ggaggcctaactggccggtacctagatctgctagcctcgaggatatcagcggccgctggcaaaatgtctgggagtcaccaaatgc agagaatgccatattcaacaaagcctgataaccaaggactcagtggactaattggcagtcctatcccagatccaaggttccttgagc caggggcaagctaggatatgctcccaggtatcttctcccttaggactttaggtttcttggccacttcctcttatttcagtgaaagcagatc cactccattgacacttgtggtcacagtctagcacgactgctcccttccttcttttctccctccctgcgcagcttcatttgctcccagtagtg gctggaaaaacaccaaattccaatccgcggtttctcccttctacttcctggaaacatccaagggctcggcacttactcagcagattca aacttccactttccatcactcatcgaggatgatgctgctccttggcaccaaccaccctgcctgactccaccctctggcttacaataaaa ggctgaggcagagccgctagaaatgcagagacacagacagaggccagcccagaaaccagccaactctcactgaaaagcttccc
Spry2-enh	ggaggcctaactggccctgaagttagggtcagtcatgtccccaggtctgaactgtcttcccttgccagccttctttgccacacattaaa atgacaggccacattcccctcactactccctcagcgccccctgtagcagggaggtctgcttaattaggcttacaggcagactgactg ggtaattcttggatcttgagagaagtactttgtattgtaggctgttggtatcacccctggcctttcccttcctattgccagcagtgaagtc caaaaaatcttaaaggccttgcctaatggaccttgaggcgaggggactagacacccaagaccttaagtagataatgttatcagtcttc

	ttccaagacttagcacctaagtctcagattatcagcttggaaagctcgaggaccatttttctctcctctctcatctgtggcatcacaattta<br>caacaataaggtgcacggtaagagtttagtcagtgacgagtagttgcaccctgggcctgaaagcttggttgtttagcagtactatgga<br>tccaacctaggctctcccacatgctaggccaccgctctacctaggagctacagcagttctactgttctcagacagttctgttcttcaca<br>gcgtagggcaaagtctcagaaggcctcctactgggctcatcgggtgacttcacagatgataagtaagaagtcagtactttctgacag<br>agctggttattttcagtgtttgtttaaggttttactcagtagctaagagtgcaacaaagtgaaaccaatcaggctttagcccaactttgct<br>ggtccagattccaggagacacctcagctaaagccaagtgtcgagggcatggctgtgtcatcttcctggagtcagtcccttacctgcg<br>gccgcaaaaggaaaa
Esm1A-enh	ggaggcctaactggcccacctccactcgctcaagtctgaagtgttttgaattgtggtggtaattctgtatgcataaagttttccgctcttg<br>tacaaattattcagttatggaacacagagactttgacagtgttctccagtcactgccctcaaaggagtgcctagaagctcgggttttcct<br>tgtttcctgttgtaatccagtcgaccactgacattagccatcatgagcatctgtgtatcgtaccagaaaggaatgtctcaagctgcagg<br>tgacagaaatgaatcacatacaggcactcacagaacaaaggaacagtattttcttccaataataaagacaatccgggaggtcttgct<br>atcttcagggcagtgattctgagtttgccatgccaggcctctagatgtccttttgtttgaaatctgtgtggaaactcccagactttgcaac<br>tcttgcgtcctttttgtgtcttatttagatttctattgccatgataaaataccttgaccaaaagcatctaagggcgaggagagggtttatttc<br>acctcatagcttgtagtccttgaagggaagtcagcaggaatttggttggaacctggagggaggcacctgggagcagggactgaag<br>cagaggccatagaggaggaaaactgcatattgacttgcttctcctggctcgctcagctggcttttcttacaacgcaggaagccctgc<br>ccaggagtgccactgcccaggagtgccactgccccccagtggactgggctctcctctatcaattagcaatcaaatgccccaggctt<br>gcctatagacgagcctctaagtctgataaatgccttttaaaaaaatcaattcctgttcagatttaactctacttgtgtcaaattaacaacaa<br>caacagcagcagtagcagcagcagccacccacctaaccagaacaacatgcatttaaaaccagcatcacatggaaaccaccagtc<br>agttgctggcttgagtgaaatctgggactcctgggaccaaagacacattgacctccacatgcttggacaactgaagatagggaaat<br>atatcctggagagggcacttcatgaccagcccagtgaagtaagacaccctagatctctagaggctagaggtgctctttctacaggta<br>gcatcttttcaaggagtttactccatatccttgagcctacagtggagcggccgcaaaaggaaaa

Enhancer	Forward Primer (5'-3')	Reverse Primer (5'-3')
Esm1B-enh	ggaggcctaactggcctacccttcctgaatctatg	ctagctagccatagtctcccctagttcc
Esm1C-enh	ggaggcctaactggccctaaaatccaagtggctttag	ctagctagcagagggtagcatgaaaacc
Egr3-enh	ggaggcctaactggccttcttcttgtccttgg	ctagctagccatctagttcagcaaagag

qRT-PCR expression lnc-Ang184 and

lncAng383 CRISPR Phenotype

Refers to Figure 5

Gene	Forward Primer (5'-3')	Reverse Primer (5'-3')
Ccl2	gcagctcagcagaggtagttgg	cggtatagggtctgagaagattaccg
Serpine1	gcagctctctgtagcacaagcc	cggcctctgttggattgtgccg

IL6	ggataccacccacaacagaccag	cgatgagttttctgacagtgcatcatc
lnc-Ang383	atgtccacactgtatgcctc	ctgctccacttctctcactatg
lnc-Ang184	cagacctagtggcagtttcc	cgcctatacctcaacacttcc
Ramp3	gcaccttctccctctgttg	tccagcattcctgtctcattg

Dicer-substrate short-interfering RNAs (DsiRNAs)

Refers to Figure 5H

**Name**	**Sense (5'-3')**	**Anti-sense (5'-3')**
Dsi-lnc-Ang383	gaacuaauagcuaaggcuuuccaaguu	cuuggaaagccuuagcuauuagutc

Refers to Supplementary Figure 10

**Gene**	**Forward Primer (5'-3')**	**Reverse Primer (5'-3')**
lnc-Ang362	gctcccattctgttccttttg	cacactcaccttcctattccc

CRISPR-sgRNAS

Refers to Figure 5 (I-M) and 9 (A-D)

**Enhancer sgRNA**[#]	**Forward Primer (5'-3')**	**Reverse Primer (5'-3')**
lnc-Ang383SG	caccgactgtttattcgttctctac	aaccgtagagaacgaataaacagtc
lnc-Ang383ASG	caccgctttgattccagcaccgaag	aacccttcggtgctggaatcaaagc
Egr2SG	caccgcgcccttcccggcgtgtct	aaccagacacgccgggaagggcgc
Egr2ASG	caccggagcgcatcggatgcgggc	aaccgcccgcatccgatgcgctcc
Fgf2SG	caccgcggctctactgcaagaacgg	aaccccgttcttgcagtagagccgc
Fgf2ASG	caccgtctgggtccataaggctacc	aaccggtagccttatggacccagac
FstSG	caccgcgactgcactctccaagcta	aacctagcttggagagtgcagtcgc
FstASG	caccgatgcgcccagaggttcggct	aaccagccgaacctctgggcgcatc

Tgif1SG	caccgatgctttaatcccatcactc	aaccgagtgatgggattaaagcatc
Tgif1ASG	caccgttccataatacccgactgta	aacctacagtcgggtattatggaac

CRISPR Genotyping

Refers to Supplementary Figure 9

**Gene**	**Forward Primer (5'-3')**	**Reverse Primer (5'-3')**
Fst	ggagcatctgctaagcgtaaa	ctccgacagataattgggaagg
Tgif1	gcattgatacatgccagcaaa	gtgcatgctatgttcgcataat
lnc-Ang383	tacagaagaagacagcacacac	agctaaggctttccaagtttaatg
Fgf2	agtccggctgcactagg	ggagggataatgctggagtttg
Egr2	ggtagagagaatggagcagaag	gtagcgtgggtcaagaaaga

qRT-PCR expression

Refers to Figure 8 A-N and Supplementary Figure 16 A-D and 18 A-D

SE associated Genes

**Gene**	**Forward Primer (5'-3')**	**Reverse Primer (5'-3')**
Fst	gagtggaagagatagggaagc	agcgagtgtgccatgaag
Egr2	gaaggtctggtttctaggcg	aatggtgaactgggaggc
Tgif1	tgacgatgaaaggtccaagg	cgaaatgatttacacgagcagg
Fgf2	acccacacgtcaaactacag	ggcgttcaaagaagaaacactc
Flt1	cctcagcctaccatcaagtg	gccctcgattctgtttcctatg
Foxp1	gttttgtgcgagtagagaacg	cattgagaggtgtgcagtagg
Brd4	ttgatgcttgagttgtgtttgg	catgatagtccaggcaaaggg
Brd2	gaagcgggaactagagaagc	gaactagaagcactgagacgg
Brd3	tcctctttcgatttggctgc	cccagacgagattgagattgac
Nudt6	ggtgctaacatcaaatacggc	ctctgggcttctgcttcc

Spata5	ccagtgctccttaccagtttg	cagctttcacacccacattc
Ado	gcctggacgatggatttactg	gcagtgacaaaaccaggaatc
Nrbf2	gggtctatggaagtgatggaag	tcagcttcatggcttcagag
Dlgap1	gccacactttgaatgctacac	agaccgaaggagaaaatgcc
Myom1	tgattacggctattcccatgg	accatgtagttgcctgacag
Ndufs4	gatcccaggcattcatcgtag	aactacaggtacacttggcag
Mocs2	tgccaagaagctctcagtg	tgccatgggtagatatgcttc

ChIP

Refers to Figure 8 O-P

SE Regions	**Forward Primer (5'-3')**	**Reverse Primer (5'-3')**
Tgif1	agtaaggttgagtcgggatttg	tgccctccacaaagtgaataa
Fgf2	gcagcagtgttcttgtctttg	gtgaggtgggcttgtttact
Foxp1	atgtggaacctctccactttac	ccacgtcctacagcattgat

Supplementary Table 1. List of oligonucleotide sequences used in this study. * *m* denotes mouse genes. # SG denotes sense guide RNA and ASG denotes anti-sense guide RNA.

GWAS chromosome coordinates	GWAS ID	PubMed ID	Authors (et al.)	Journal	Trait	Genes	hg19 co-ordinates	Rat enhancer co-ordinates
chr9_22115958 _22115959	rs2383207	20622881	Gretarsdottir S	Nat Genet	Abdominal aortic aneurysm	CKDN2B, CDKN2A	chr9_22114267 _22116382	chr5_109036837 _109039175
chr5_39397131 _39397132	rs1195992 8	20383146	Kottgen A	Nat Genet	Chronic kidney disease	DAB2, C9	chr5_39397114 _39401158	chr2_55707758_ 55711091
chr1_15095147 6_150951477	rs267734	20383146	Kottgen A	Nat Genet	Chronic kidney disease	SETDB1, LASS2, ANXA9, PRUNE, FAM63A, BNIPL	chr1_15094951 5_150952124	chr2_190219397 _190221430
chr21_3559382 6_35593827	rs2845106 4	26343387	Nikpay M	Nat Genet	Coronary artery disease	KCNE2	chr21_3559306 4_35594753	chr11_32159399 _32162443
chr9_2211528_ 22115286	rs944797	21971053	Takeuchi F	Eur J Hum	Coronary heart disease	CDKN2A, CDKN2B	chr9_22114267 7_22116382	chr5_109036837 _109039175

				Genet				
chr2_62713532_62713533	rs6545946	22412388	Kenny EE	PLoS Genet	Crohn's disease	TMEM17, EHBP1, CPAMD8, AK3	chr2_62712712_62716292	chr14_103197611_103200563
chr1_83220655_83220656	rs11582609	26305897	Iyengar SK	PLoS Genet	Diabetic kidney disease	intergenic	chr1_83217751_83221886	chr2_246266690_246271012
chr20_10965997_10965998	rs1887320	25249183	Lu X	Hum Mol Genet	Diastolic blood pressure	JAG1	chr20_10957868_10966395	chr3_125541543_125548196
chr9_22115104_22115105	rs10965235	20601957	Uno S	Nat Genet	Endometriosis	CDKN2BAS	chr9_22114267_22116382	chr5_109036837_109039175
chr1_8150637_8150638	rs72634258	26192919	Liu JZ	Nat Genet	Inflammatory bowel disease	NR	chr1_8149972_8152812	chr5_167965360_167968601
chr9_22114468_22114469	rs10733376	25256182	Foroud T	Stroke	Intracranial aneurysm	CDKN2BAS, ANRIL	chr9_22114267_22116382	chr5_109036837_109039175
chr21_35593826_35593827	rs28451064	26343387	Nikpay M	Nat Genet	Myocardial infarction	KCNE2	chr21_35593064_35594753	chr11_32159399_32162443

chr13_102145559_102145560	rs1335587	23251661	Comuzzie AG	PLoS One	Obesity-related traits	ITGBL1	chr13_102123151_102146733	chr15_108803525_108814397
chr10_44458713_44458714	rs898549	23251661	Comuzzie AG	PLoS One	Obesity-related traits	C10orf136	chr10_44457081_44461770	chr4_153840182_153845918
chr14_94972241_94972242	rs11627075	23251661	Comuzzie AG	PLoS One	Obesity-related traits	SERPINA12	chr14_94972049_94977481	chr6_128102375_128105662
chr5_16913854_16913855	rs17614462	23251661	Comuzzie AG	PLoS One	Obesity-related traits	MYO10	chr5_16901265_16914607	chr2_77201740_77210481
chr5_131430117_131430118	rs657075	24390342	Okada Y	Nature	Rheumatoid arthritis	CSF2, IL3	chr5_131429842_131432784	chr10_39641675_39644527
chr5_131430117_131430118	rs657075	22446963	Okada Y	Nat Genet	Rheumatoid arthritis	CSF2	chr5_131429842_131432784	chr10_39641675_39644527
chr10_63785088_63785089	rs10821944	22446963	Okada Y	Nat Genet	Rheumatoid arthritis	ARID5B	chr10_63784082_63790216	chr20_21238868_21245054

chr1_205509297_205509298	rs181502228	26606652	Alarcon-Riquelme ME	Arthritis Rheumatol	Systemic lupus erythematosus	DSTYK	chr1_205506229_205515389	chr13_44982874_44986847
chr20_10965997_10965998	rs1887320	25249183	Lu X	Hum Mol Genet	Systolic blood pressure	JAG1	chr20_10957868_10966395	chr3_125541543_125548196
chr2_12640740_12640741	rs1534422	19430480	Barrett JC	Nat Genet	Type 1 diabetes	intergenic	chr2_12629402_12645547	chr6_39702585_39708724
chr22_30581721_30581722	rs5753037	19430480	Barrett JC	Nat Genet	Type 1 diabetes	intergenic	chr22_30581369_30583153	chr14_84958839_84960817
chr10_71452284_71452285	rs2812533	24509480	Mahajan A	Nat Genet	Type 2 diabetes	C10orf35	chr10_71447476_71452665	chr20_29456788_29457999

Supplementary Table 2: GWAS SNPs associated with AngII regulated enhancers. GWAS SNPs present in human sequences orthologous to rat enhancers were identified. Only diabetes and CVD associated SNPs are shown.
